# Identification of domestication-related loci associated with flowering time and seed size in soybean with the RAD-seq genotyping method

**DOI:** 10.1038/srep09350

**Published:** 2015-03-23

**Authors:** Ling Zhou, Shi-Bo Wang, Jianbo Jian, Qing-Chun Geng, Jia Wen, Qijian Song, Zhenzhen Wu, Guang-Jun Li, Yu-Qin Liu, Jim M. Dunwell, Jin Zhang, Jian-Ying Feng, Yuan Niu, Li Zhang, Wen-Long Ren, Yuan-Ming Zhang

**Affiliations:** 1 State Key Laboratory of Crop Genetics and Germplasm Enhancement, National Center for Soybean Improvement, Jiangsu Collaborative Innovation Center for Modern Crop Production, Nanjing Agricultural University, Nanjing 210095, China; 2 BGI-Shenzhen, Shenzhen 518083, China; 3 Soybean Genomics and Improvement Laboratory, Agricultural Research Service, United States Department of Agriculture, Beltsville, Maryland 20705, USA; 4 College of Life Science, Linyi University, Linyi 276005, China; 5 Crop Research Institute, Linyi Academy of Agricultural Sciences, Linyi 276012, China; 6 School of Agriculture, Policy and Development, University of Reading, Reading RG6 6AR, United Kingdom; 7 Statistical Genomics Lab, College of Plant Science and Technology, Huazhong Agricultural University, Wuhan 430070, China

## Abstract

Flowering time and seed size are traits related to domestication. However, identification of domestication-related loci/genes of controlling the traits in soybean is rarely reported. In this study, we identified a total of 48 domestication-related loci based on RAD-seq genotyping of a natural population comprising 286 accessions. Among these, four on chromosome 12 and additional two on chromosomes 11 and 15 were associated with flowering time, and four on chromosomes 11 and 16 were associated with seed size. Of the five genes associated with flowering time and the three genes associated with seed size, three genes *Glyma11g18720*, *Glyma11g15480* and *Glyma15g35080* were homologous to Arabidopsis genes, additional five genes were found for the first time to be associated with these two traits. *Glyma11g18720* and *Glyma05g28130* were co-expressed with five genes homologous to flowering time genes in Arabidopsis, and *Glyma11g15480* was co-expressed with 24 genes homologous to seed development genes in Arabidopsis. This study indicates that integration of population divergence analysis, genome-wide association study and expression analysis is an efficient approach to identify candidate domestication-related genes.

The transitions from vegetative to reproductive growth (days to flowering) and from generation to generation through seeds are important stages of the plant life cycle. Flowering time reflects the adaptation of a plant to its environment, and the time required to mature varies widely among cultivars^1^. Seeds are important in the reproduction and spread of flowering plants, and seed size partly reflects the efficiency of plant production^2^. Both flowering time and seed size are important traits involved in domestication, a process accompanied by reduction in genetic diversity and loss of important traits preserved in wild relatives.

Morphological, physiological and molecular markers have been widely used to measure the genetic diversity of wild and cultivated plants in rice^3^, soybean^4^, and wheat^5^, to deduce the geographic regions of domestication, and to screen for breeding material. Linkage and association studies have identified quantitative trait loci (QTL) associated with domestication-related (DR) traits in various plants^6^,^7^and animals^8^, such as the lateral branching locus *teosinte branched1* (*tb1*) in maize^9^, the seed shattering locus *qSH1* in rice^10^, and the milk-production locus *DGAT1* in dairy cattle^11^. Numerous DR genes have also been identified, including *GW8* in rice^12^, *Q* in wheat^13^, *vrs1* in barley^14^, and *RYR1* in pig^15^. As whole genome sequences from almost all major plants have become available, substantial progress has been achieved, including 1) Hundreds of DR genes have been identified by comparative analysis of genomes in plants^16^ and animals^17^; 2) Candidate genes resulting from selection were detected by comparative analysis and functional tests^7^, for example, the genes *LOC_Os05g20290* and *LOC_Os08g40710* in rice^18^ and the genes *GRMZM2G448355* and *abph1* (*GRMZM2G035688*) in maize^19^; and 3) The molecular mechanisms underlying DR traits have been reported^20^,^21^. In addition, the ancient DNA and genomes of fossils have contributed to the histories of domestication and evolution in horses^22^, cattle^23^, and *Arabidopsis thaliana*^24^. However, little is known about the loci/genes underlying DR traits in soybean.

The soybean plant originated in China and was first domesticated by Chinese farmers between 6,000 and 9,000 years ago^25^. The modern domesticated soybean (*Glycine max*) is an economically important crop because it has high protein and oil contents, can fix nitrogen through microorganisms in the soil^26^,^27^, and is a model plant for legume research. The cultivated soybean (*G. max*) differs from the wild soybean (*G. soja*) in many traits, for example, the cultivated soybean forms flowers earlier and has larger seeds than the wild soybean^28^. Previous research has led to the discovery of many QTL correlated to DR traits such as seed size^29^,^30^,^31^,^32^,^33^ and other traits (http://www.SoyBase.org). Some genes controlling DR traits in soybean have also been discovered, e.g., *E1–E4*^34^,^35^,^36^,^37^, *FLC*, *VRNA*, *ELF8*, *PHYE*, *PHYA*^16^, *CDF3*, *VRN1*, *SVP*, *AP3* and *PIF3* for flowering time^38^, and *Dt1* for determinate growth habit^39^. Although the genes and their molecular mechanisms for some DR traits in soybean have been investigated^38^,^39^, the genes/loci underlying many other DR traits remain to be addressed.

In this study, restriction-site-associated DNA (RAD) tags from 14 wild, 153 landrace, and 119 bred accessions were sequenced, and the sequence variants were analyzed to detect DR loci by testing the independence between the SNPs and soybean evolutionary classes (wild, landrace, and bred) and comparing the genetic diversity between the wild and cultivated soybeans. Genome-wide association of the detected DR loci with DR traits (flowering time and seed size) were also studied. Candidate genes predicted to be involved in these two traits were pinpointed using comparative genomics technology. Co-expression analysis for individual candidate genes was also conducted.

## Results and Discussion

### Phenotypic characteristics of flowering time and seed size

Flowering time was measured by the days from the date of emergency to the date of first and full flowering in this study. The average plus standard error were 51.83 ± 3.73, 46.22 ± 0.83 and 35.60 ± 0.64 (days) for first flower and 55.95 ± 4.00, 49.96 ± 0.86 and 38.57 ± 0.65 (days) for full flower in wild, landrace and bred soybeans in 2010–2012, respectively. *G. soja* flowered later than *G. max*.

Seed size was characterized by seed length (SL), seed width (SW) and 100-seed weight (100 SW) in this study. The averages plus standard errors in wild, landrace and bred soybeans in 2008–2012 were 4.76 ± 0.18, 7.61 ± 0.08 and 7.85 ± 0.06 (mm) for SL, 3.56 ± 0.17, 6.54 ± 0.07 and 6.63 ± 0.05 (mm) for SW, and 2.99 ± 0.45, 15.43 ± 0.48 and 16.85 ± 0.39 (g) for 100 SW, respectively, indicating much smaller seeds in *G. soja* than in *G. max*.

Distinct difference of these traits between *G. soja* and *G. max* suggested that these traits are domestication-related. Flowering time and seed size were also considered as DR traits in other reports^25^,^28^,^40^,^41^,^42^, although domestication traits in soybean include more than these two traits, such as indeterminate habit and pod dehiscence.

### Detection and distribution of domestication-associated loci

**Figure 1:**
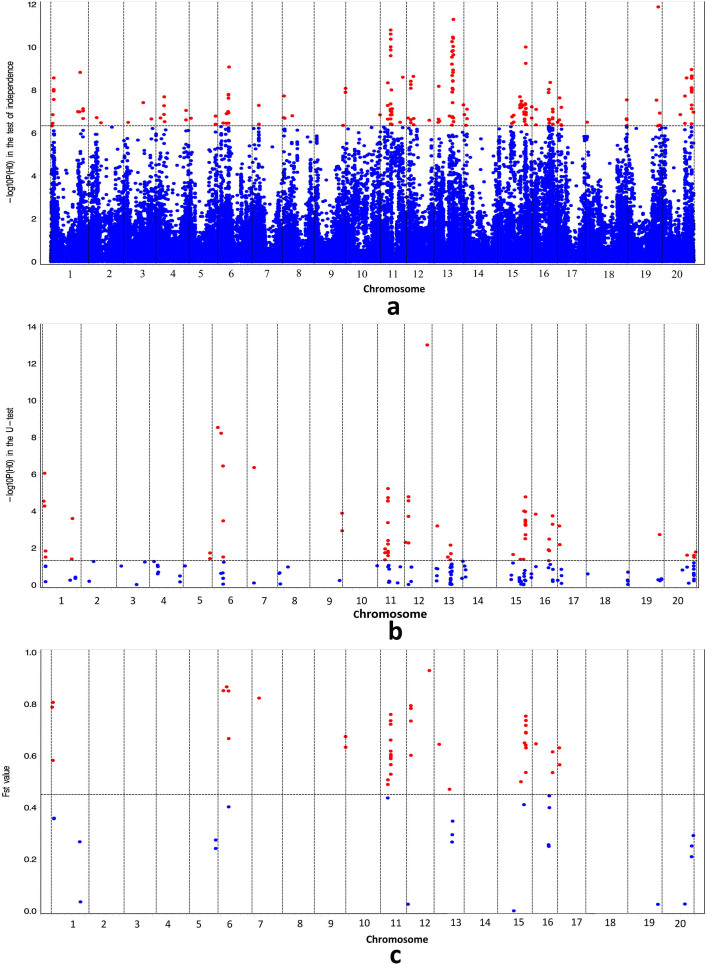
Detection of domestication-related loci in the soybean genome using *χ*^2^ test of independence (a), U-test (b) and genetic diversity analysis (c). Significances of all the SNPs in the above three analyses were marked by red (significant) and blue (not significant) dots. The genomic positions of SNPs on chromosomes 1 to 20 were 7166 ~ 55874947, 2395 ~ 51643854, 45230 ~ 47773436, 15992 ~ 49228296, 9016 ~ 41933701, 1142 ~ 50641309, 37534 ~ 44659030, 3583 ~ 46944564, 25976 ~ 46841908, 24539 ~ 50962464, 30293 ~ 39163227, 13701 ~ 40093893, 10047 ~ 44402574, 13165 ~ 49710404, 8319 ~ 50879005, 14132 ~ 37370388, 47058 ~ 41905331, 426 ~ 62303776, 9282 ~ 50584403 and 18252 ~ 46703751 (bp), respectively.

Based on the sequence obtained from 286 accessions through the RAD-seq genotyping approach, a total of 106,013 SNPs were identified. *χ*^2^ tests of independence between the SNPs and evolutionary classes (wild, landrace, and bred) of soybean showed that 198 SNPs were significant at P-value ≤ 4.72 × 10^−7^ (Fig. 1a). A U-test determined that the allelic frequency for 72 of the 198 SNPs were significantly different between the wild and cultivated classes (Fig. 1b). The fixation index (F_ST_) analysis demonstrated that 48 of the 72 SNPs had an F_ST_ value greater than 0.45 (Fig. 1c; Table S1).

Of the 48 DR loci, 3, 4, 1, 2, 14, 5, 2, 11, 3 and 3 loci were located on chromosomes 1, 6, 7, 9, 11, 12, 13, 15, 16 and 17, respectively (Fig. 1c). However, 52% of the 48 loci were located on chromosomes 11 or 15, and these two chromosomes harbour more DR loci than other chromosomes. Similar results were also observed by Kim *et al*.^43^, Chung *et al*.^44^ and Li *et al*.^45^. On the two chromosomes, Kim *et al*.^43^ identified four DR genes (*Glyma15g14980*, *Glyma15g23400*, *Glyma11g05720* and *Glyma15g04930*) that were associated with flowering time; Chung *et al*.^44^ detected 204 soybean candidate DR genes; and Li *et al*.^45^ found three DR genes (*Glyma11g03340*, *Glyma11g03330* and *Glyma11g03320*) near the reported flower colour QTL.

A total of 12 DR loci were identified in 11 genes with 4 loci in the coding regions of the genes. Of the 11 genes whose functions were previously annotated in the Pfam and NCBI non-redundant databases, three and two genes were located on chromosomes 11 and 15, respectively. The common allele for each of the 12 loci was different in the bred and wild soybean, and common alleles for each of the 3 DR loci on chromosome 11 were the same in both the landrace and wild soybeans.

### Genome-wide association studies (GWAS) for flowering time and seed size

**Figure 2:**
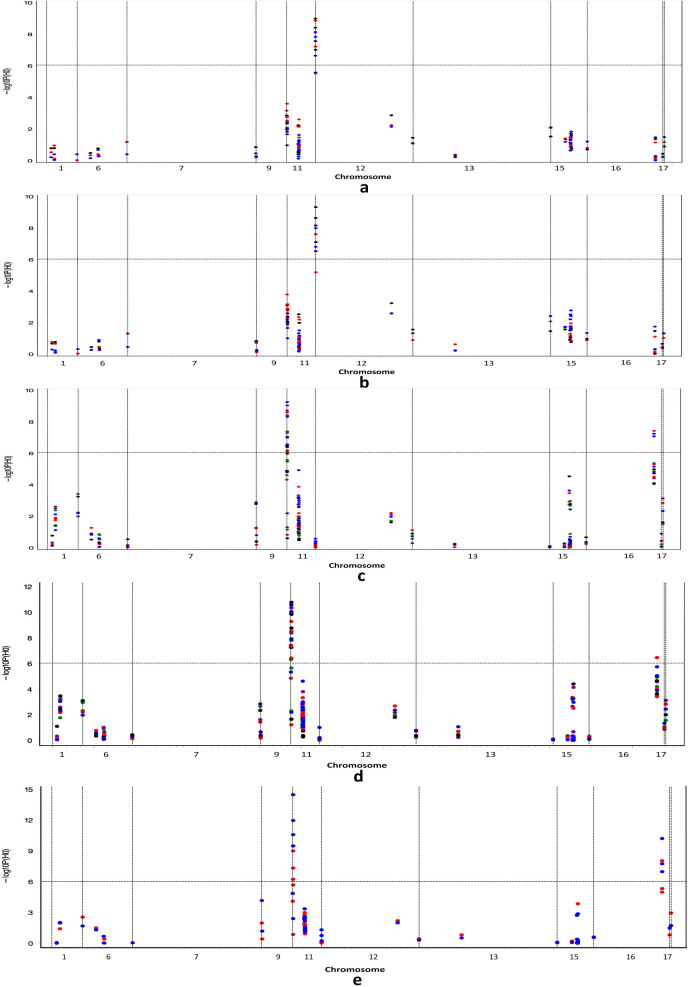
Genome-wide association of first (a) and full (b) flowering times, seed length (c), seed width (d) and 100-seed weight (e) in soybean during 2008–2012 with domestication-related SNPs on chromosomes 1, 6, 7, 9, 11, 12, 13, 15, 16 and 17. Dots with green, purple, black, red, and blue colors denote 2008, 2009, 2010, 2011, and 2012, respectively.

GWAS for flowering time (first and full) and seed size (SL, SW and 100 SW) was performed based on a dataset containing 286 accessions genotyped with 55,052 SNPs (Fig. S1). Because our purpose was to investigate the association of the DR loci with the two DR traits, we only analyzed the association of the DR loci listed in Fig. 2 and Table 1.

Four QTL were associated with flowering time (P-values of 4.68E-10 to 2.95E-7), and each QTL explained 5.32–7.32% of the total phenotypic variance. These QTL were positioned at 5895633, 5895655, 5895785, and 5957099 (bp) on chromosome 12 (Fig. 2a, b). The first three loci were associated with the first and full flowering times in 2010 to 2012, while the last locus was associated with the first flowering time in 2010 and 2012 and with the full flowering time in 2012. In addition, two loci at genomic positions 15362036 bp (P-values of 0.0064 to 0.0234) on chromosome 11 and 39607166 bp (P-values of 0.0183 to 0.0434) on chromosome 15 were associated with the flowering times at a 0.05 probability level in 2010 to 2012, except for the fact that the later locus was almost associated with the first flower at a 0.05 probability level in 2012 (P-value = 0.0640) (Fig. 2a, b).

A total of 8 QTL, each explaining 0.91–16.85% of the total phenotypic variance of seed size, were identified (P-values of 3.24E-15 to 7.76E-7). These QTL were mapped to genomic positions 10957940, 11100801, 11120981, 11121256, and 11121307 bp on chromosome 11 and 30207191, 30207175, and 30210113 bp on chromosome 16 (Fig. 2c–e). Among the five SNP loci on chromosome 11, the last four loci, which were close to the DR locus at 11111516 bp (~10 kb), were associated with the three seed size traits. The DR locus at 10957940 bp was associated with SW in 2008 to 2010 and with SL in 2009. Among the three loci on chromosome 16, one locus at genomic position 30207191 bp, which was close to the DR locus 30207175 bp, was correlated with SL and 100 SW in 2011 and 2012 and with SW in 2011. The DR locus at 30207175 bp was associated with 100 SW in 2012, and the DR locus at genomic position 30210113 bp was associated with SL and 100 SW in 2012. In summary, all four DR loci were associated with seed size traits.

### Candidate DR genes for flowering time

**Figure 3:**
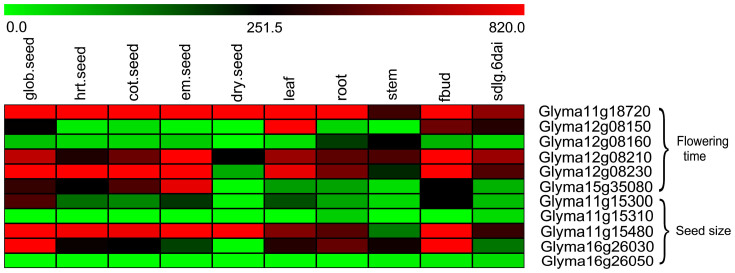
Expression levels of candidate genes for soybean flowering time and seed size in various developmental stages or tissues. The expression data were extracted from the soybean transcriptome data deposited at NCBI (http://www.ncbi.nlm.nih.gov/geo/).

Candidate DR genes were selected if: 1) genes containing DR loci or genes in the adjacent regions to which DR loci were significantly associated with the flowering time or seed size, and 2) genes with high level of transcriptome expression. The locus at 15362036 bp on chromosome 11 was in the gene *Glyma11g18720*, which was homologous to the gene *OTLD1* regulating *FLC* for flowering time and seed germination in Arabidopsis^46^,^47^,^48^ (Table 1, Fig. S2). The locus at 39607166 bp on chromosome 15 was in the gene *Glyma15g35080* which regulated flowering time in soybean^38^. We also observed a relatively high expression of the *Glyma11g18720* and *Glyma15g35080* at specific stages of flowering time and seed development (Fig. 3).

A multiple linear regression analysis of each selected soybean gene (*y*) on *Glyma11g18720* (*x*_1_) and *Glyma05g28130* (*x*_2_) (homologous to *FLC*, Fig. S3) based on expression data, with the model *y* = *b*_0_ + *b*_1_*x*_1_ + *b*_2_*x*_2_ + *b*_12_*x*_1_*x*_2_ + *ε*, identified five significant flowering-time-related genes *Glyma06g02470*, *Glyma08g02490*, *Glyma11g02110*, *Glyma15g17480*, and *Glyma02g11060* with P-values of 1.28E-5 to 7.81E-2 (Table 2). Of the five genes, *Glyma06g02470* was homologous to the genes *EEL* and *AREB3*^38^ related to flowering time in Arabidopsis (Fig. S4); *Glyma08g02490* to the genes *SPA1*–*SPA4*^38^ (Fig. S5); *Glyma15g17480* to the genes *FKF1*, *LKP2*, and *ZTL*^38^ (Fig. S6); and *Glyma02g11060* to the genes *TEM2*, *RAV1*, *TEM1*, and *EDF3*^38^ (Fig. S7).

The genes *Glyma12g08150* and *Glyma12g08160* were adjacent to the domestication regions of 5895633 to 5895785 bp on chromosome 12, and the genes *Glyma12g08210* and *Glyma12g08230* were in close proximity to the DR locus at 5957099 bp on chromosome 12. The likely association of these genes with domestication was also reported by Chung *et al*.^44^. In addition, based on the soybean transcriptome data deposited at NCBI (http://www.ncbi.nlm.nih.gov/geo/), all the genes had relatively high expression levels in floral bud tissue (Fig. 3). Therefore, the above 10 genes may be associated with flowering time.

### Candidate DR genes for seed size

The genes *Glyma11g15300* and *Glyma11g15310* were close to the DR locus at 10957940 bp on chromosome 11. *Glyma11g15480* contained a DR locus at 11111516 bp on chromosome 11, and *Glyma16g26030* and *Glyma16g26050* were close to the domestication regions of 30207175 to 30210113 bp on chromosome 16. The three genes *Glyma11g15300*, *Glyma11g15480* and *Glyma16g26030* had relatively higher expression levels during seed development than did *Glyma11g15310* and *Glyma16g26050* (Fig. 3). The soybean gene *Glyma11g15480* is homologous to the Arabidopsis gene *NOT2A*^49^ (Fig. S8) and was found to be associated with seed size in this study. A correlation analysis of *Glyma11g15480* with each selected soybean gene based on the gene expression dataset showed that 24 genes were significantly correlated with *Glyma11g15480* (P-values of 1.00E-4 to 4.90E-2) (Table 2). Among the 24 genes, 11 were homologous to five epigenetic regulation endosperm genes in Arabidopsis (*FIS2*, *MSI1*, *FIE/FIS3*, *DDM1*, and *MET1*)^50^,^51^,^52^ (Figures S9–S13); six were homologous to four endosperm development genes (*AP2*, *AGL61*, *SHB1*, and *EMS1*)^50^,^53^,^54^,^55^ (Figures S14–S17); five were homologous to two integument development genes (*ARF2/MNT*, and *TTG2*)^56^,^57^ (Figures S18–S19); and two genes were homologous to the embryo development gene (*ANT*)^58^ (Fig. S20). Therefore, the above 27 genes may be associated with seed size.

In addition, significant difference of allele frequencies for the SNPs in coding region of the above eight candidate DR genes in GWAS was observed between wild and cultivated soybeans (Tables S2, S3). Although the number of wild accessions was smaller than the number of cultivated soybeans, it is close to that reported in Chung *et al*.^44^ and Lam *et al*.^59^, and we adopted a stringent threshold of statistical significance in determining domestication loci (*α* = 4.72E-7 and F_ST_ > 0.45) and GWAS (*α* = 9.08E-7).

Some QTL (or genes) associated with DTs in this study were consistent with those reported previously (Table S4), for example, *Glyma12g08150*, *Glyma12g08210* and *Glyma12g08230* were reported as candidate domestication genes by Chung *et al*.^44^.

## Conclusion

A total of 48 DR loci in the soybean genome were identified. Most of these loci were on chromosomes 11 and 15. Among these DR loci, 10 loci were associated with flowering time or seed size. Eight genes near the 10 loci were associated with the two traits. Among the eight genes, three genes *Glyma11g18720*, *Glyma11g15480* and *Glyma15g35080* were homologous to Arabidopsis genes, three known DR genes *Glyma12g08150*, *Glyma12g08210* and *Glyma12g08230* were linked to flowering time; and the other two DR genes *Glyma11g15300* and *Glyma16g26030* were reported for the first time. *Glyma11g18720* and *Glyma05g28130* were co-expressed with five genes homologous to flowering time genes in Arabidopsis, and *Glyma11g15480* was co-expressed with 24 genes homologous to seed development genes in Arabidopsis. The method that integrates domestication analysis, GWAS and gene expression analysis is an efficient approach for identifying potentially useful DR genes.

## Methods

### Germplasm for genome-wide re-sequencing

The 286 soybean accessions used here included 14 wild, 153 landrace, and 119 bred accessions that were randomly selected from 6 geographic regions in China using a stratified random sampling method (Table S5). The seeds of the accessions were obtained from the National Centre for Soybean Improvement and Linyi Academy of Agricultural Sciences, and planted in three-row plots in a completely randomised design at the Jiangpu Experimental Station of Nanjing Agricultural University during 2008–2012. The plots were 1.5 m wide and 2 m long. A total of 20 seeds from five plants of the middle row in each plot were randomly selected for the measurement of the seed size traits, including SL and SW, using digital verniercalipers. The SL and SW for each accession were averaged based on 20 seeds and 100 SW for each replicate. The first and full flowering dates for each accession were recorded in the field during 2010 and 2012.

### Genome-wide re-sequencing and sequence alignment

Approximately 0.3 g of fresh leaves obtained from each accession in 2012 was used to extract genomic DNA using the cetyltrimethylammonium bromide method. The DNA was digested with the *EcoRI* (G^∧^AATTC) enzyme. The RAD-seq libraries for sequencing were prepared according to the protocols described by Baird *et al*.^60^. We sequenced 50 bp at each end and used SolexaPipeline 1.0 for base call of 50-bp reads from the raw fluorescent images. To ensure quality, the raw sequence data were processed in two steps. In the first step, reads with incorrect adapter sequences were deleted, and any reads containing more than 50% low quality bases (quality value < = 5) were removed. In the second step, the remaining reads were demultiplexed according to the index of each sample. The sequences were subsequently aligned on the soybean reference genome Glyma1.1 (http://www.jgi.doe.gov) using the Burrows-Wheeler Alignment Tool (BWA)^61^. The base recalibration and determination of the SNP allele were performed using GATK^62^.

### Detection of DR loci

The independence between each SNP and the evolutionary class (wild, landrace, and bred) was determined using the χ^2^ test^63^. The significant threshold *α* for each test was adjusted using the Bonferroni correction, that is, *α* = 0.05/106,013 = 4.72 × 10^−7^ (106,013 was the total number of SNPs identified). The SNPs significantly associated with evolutionary classes were used for the subsequent analysis.

A U-test at the 0.05 significance level was used to test the significance of the allelic differences between the wild (14) and cultivated (272) accessions for each candidate SNP.

For all the SNPs with significant differences in the U-test, the fixation index (F_ST_) was calculated for the purpose of measuring population differentiation between the wild (14) and bred (119) classes using the software Genepop v4.2^64^, and a F_ST_ threshold value of 0.45 from Lam *et al*.^59^ were adopted for the identification of DR loci.

### Population structure analysis

The STRUCTURE 2.2 software was used to investigate the population structures based on all available accessions^65^. The number of subpopulations (*K*) was set from 2 to 7^32^. In this study, the Δ*K* method of Evanno *et al*.^66^ was used to determine the optimal value of *K*. The *Q* matrix was calculated based on inferred K.

### Genome-wide association study

A GWAS was performed using the general linear model in TASSEL V4.3 (www.maizegenetics.net/tassel) with the total average and population structure as covariates. The Manhattan plot of −log_10_*P* was generated using SAS software. As P-values from the software were not corrected for multiple test, the significance level *α* for each test was determined after Bonferroni correction, *α* = 0.05/55,052 = 9.08E-7 (55,052 was the number of SNPs with the concordance rate <99% by the TASSEL software). Elimination of the SNPs with concordance rate >99% was conducted by the TASSEL software.

### Identification of soybean DR genes and homologous Arabidopsis genes

Many genes have been annotated with high confidence in the soybean genome^26^. The most likely functions of the genes have also been determined based on the annotation of the Arabidopsis homologs. The protein sequences of the annotated genes in soybean (Glyma1.1) and Arabidopsis (TAIR9 release) were downloaded from the Phytozome (www.phytozome.net) and The Arabidopsis Information Resource (TAIR) (www.arabidopsis.org) websites, respectively.

The Pfam gene annotations were downloaded from the Phytozome database version 9.0 (http://www.phytozome.net/). The annotations of the DR genes using BLASTX (e-value lE-4 or better) against NCBI's non-redundant database have been described at http://www.ncbi.nlm.nih.gov/. The top best hits from the species of interest were extracted for each gene.

### Transcriptional activity and gene expression data analysis

The transcriptome sequences were obtained from the Gene Expression Omnibus database at http://www.ncbi.nlm.nih.gov/geo/ss (accession number GSE29163), and were used to identify the expression level of soybean gene during seed development and throughout the life cycle.

## Author Contributions

Y.-M.Z. designed the project. L.Z., S.-B.W., Q.-C.G., J.W., G.-J.L., Y.-Q.L., J.Z., J.-Y.F., Y.N., L.Z., W.-L.R. and Y.-M.Z. performed the experiments and analyzed the data. J.J. and Z.W. conducted DNA sequencing of all the accessions and analyzed the genetic diversity. Y.-Q.L. provided partly soybean materials. L.Z. prepared figures and tables. Y.-M.Z., L.Z., Q.S. and J.M.D. wrote the manuscript text. All authors reviewed the manuscript.

## Supplementary Material

Supplementary Information Supplementary information

## Figures and Tables

**Table 1:** Genome-wide association of flowering time (FT) and seed size (SS) traits with domestication-related SNPs (bold) in soybean

SNP		Domestication analysis			Genome-wide association study							Homologous gene in *Arabidopsis*	
Chr.	Position (bp)	*χ*^2^ (−log_10_*P*)	*U* (−log_10_*P*)	F_ST_	Trait	Allele	Frequency	Effect	−log_10_*P*	*r*^2^ (%)	Candidate gene	Gene	Reference
**11**	**10957940**	30.60(6.64)	−2.36(1.74)	0.4890	SS	T	0.45	−9.12 ~ −5.84	6.32 ~ 7.43	6.24 ~ 11.80	*Glyma11g15300 Glyma11g15310*		
11	11100801				SS	G	0.58	−2.10 ~ −0.58	6.52 ~ 14.49	8.14 ~ 16.26	*Glyma11g15480*	*NOT2A* (AT1G07705)	49
**11**	**11111516**	38.40(8.34)	−2.53(1.95)	0.5069	SS	C	0.38	0.90 ~ 3.90	0.60 ~ 2.40	0.91 ~ 3.20	*Glyma11g15480*	*NOT2A* (AT1G07705)	49
11	11120981				SS	C	0.53	−23.59 ~ −18.40	6.19 ~ 10.59	6.50 ~ 14.42	*Glyma11g15480*	*NOT2A* (AT1G07705)	49
11	11121256				SS	G	0.51	4.40 ~ 18.40	6.11 ~ 9.91	5.96 ~ 16.85	*Glyma11g15480*	*NOT2A* (AT1G07705)	49
11	11121307				SS	T	0.56	−0.07 ~ 4.87	6.40 ~ 11.97	6.56 ~ 16.26	*Glyma11g15480*	*NOT2A* (AT1G07705)	49
**11**	**15362036**	45.45(9.87)	−2.43(1.82)	0.5656	FT	A	0.42	−10.11 ~ −4.87	1.51 ~ 2.61	1.32 ~ 2.17	*Glyma11g18720*	*OTLD1* (AT2G27350)	46,47,48
**12**	**5895633**	37.23(8.08)	−3.73(3.71)	0.7344	FT	C	0.53	−12.96 ~ −5.21	7.63 ~ 8.65	6.16 ~ 7.00	*Glyma12g08150 Glyma12g08160*		
**12**	**5895655**	38.04(8.26)	−4.30(4.78)	0.7830	FT	A	0.52	−13.05 ~ −5.20	6.53 ~ 7.61	5.32 ~ 6.34	*Glyma12g08150 Glyma12g08160*		
**12**	**5895785**	38.74(8.41)	−4.19(4.55)	0.7938	FT	C	0.53	−4.54 ~ −1.30	8.14 ~ 9.33	6.44 ~ 7.32	*Glyma12g08150 Glyma12g08160*		
**12**	**5957099**	30.47(6.62)	−2.78(2.27)	0.6017	FT	A	0.55	−12.93 ~ −9.17	6.54 ~ 7.11	5.61 ~ 5.92	*Glyma12g08210 Glyma12g08230*		
**15**	**39607166**	32.10(6.97)	−3.89(3.40)	0.6496	FT	A	0.48	−14.97 ~ −10.93	1.11 ~ 1.74	1.06 ~ 1.54	*Glyma15g35080*	*AREB3* (AT3G56850)	38
**16**	**30207175**	30.67(6.66)	−3.47(3.28)	0.5348	SS	G	0.35	−7.48 ~ −3.97	6.99	8.49	*Glyma16g26030 Glyma16g26050*		
16	30207191				SS	T	0.44	−10.29 ~ −8.41	6.47 ~ 8.05	6.02 ~ 9.97	*Glyma16g26030 Glyma16g26050*		
**16**	**30210113**	30.90(6.71)	−3.74(3.74)	0.6148	SS	C	0.37	−2.13 ~ 0.84	7.25 ~ 10.22	7.24 ~ 12.01	*Glyma16g26030 Glyma16g26050*		

**Table 2:** Co-expressional genes of *Glyma11g18720* and *Glyma05g28130* for flowering time (FT) and *Glyma11g15480* for seed size (SS) and homologous genes in *Arabidopsis*

	Co-expressional analysis			Homologous gene in *Arabidopsis*					
Trait	Gene	F(P-value)	R^2^(%)	Gene	Similarity (%)	Notation	Functional description	Reference	Evolutionaryrelationship
FT	*Glyma06g02470*	3.77(7.81E-2)	86.91	*AT3G56850*; *AT2G41070*	23.2; 12.3	*AREB3*; *EEL*	DNA binding/transcription factor	38	Figure S4
	*Glyma08g02490*; *Glyma11g02110*	6.42(2.66E-2)	76.24;87.75	*AT2G46340; AT4G11110*	35.4–61.7	*SPA1* ~ *SPA4*	suppress photomorphogenesis, light signaling pathway	38	Figure S5
		14.33(3.83E-3)		*AT3G15354; AT1G53090*					
	*Glyma15g17480*	108.86(1.28E-5)	98.20	*AT1G68050; AT2G18915*	68.2–85.3	*FKF1*, *LKP2*, *ZTL*	control flowering time by influencing the circadian clock period, light signaling pathway	38	Figure S6
				*AT5G57360*					
	*Glyma02g11060*	13.29(4.66E-3)	86.91	*AT1G68840; AT1G13260*	53.9–61.6	*TEM2*, *RAV1*	repress flowering, light signaling pathway	38	Figure S7
				*AT1G25560; AT3G25730*		*TEM1*, *EDF3*			
SS	*Glyma01g41460*	43.85(2.00E-4)	84.57	*AT2G35670*	29.4	*FIS2*	female gametophyte, endosperm	50,51	Figure S9
	*Glyma05g26150; Glyma08g09090*	12.09–82.46	66.17–91.16	*AT5G58230*	63.0–92.5	*MSI1*	female gametophyte, embryo	50,51	Figure S10
	*Glyma11g09700; Glyma12g03700*	(<1.00E-4–8.40E-3)							
	*Glyma10g02690; Glyma02g17110*	6.40–16.87	44.43–67.83	*AT3G20740*	83.7–86.7	*FIE*/*FIS3*	female gametophyte, endosperm, embryo	50,51	Figure S11
	*Glyma13g36310*	(3.40E-3–3.53E-2)							
	*Glyma01g38150; Glyma11g07220*	7.40(2.62E-2)	48.07;46.66	*AT5G66750*	76.2; 75.5	*DDM1*	chromatin remodeling factor; embryo, endosperm	50,52	Figure S12
		6.70(2.95E-2)							
	*Glyma06g18790*	6.42(3.51E-2)	44.51	*AT5G49160*	73.5	*MET1*	methyl transferase; embryo, endosperm	50,52	Figure S13
	*Glyma11g05720; Glyma17g18640*	9.85(1.38E-2)	55.18;47.85	*AT4G36920*	46.3; 49.5	*AP2*	AP2 domain transcription factor; Integument, endosperm, embryo	53	Figure S14
		7.34(2.67E-2)							
	*Glyma20g27320*	5.37(4.90E-2)	40.16	*AT2G24840*	42.0	*AGL61*	MADS-box transcription factor; central cell, endosperm	50	Figure S15
	*Glyma07g35520; Glyma09g37000*	35.83(3.00E-4)	81.75;48.29	*AT4G25350*	68.9; 64.8	*SHB1*	Transcription co-activator; embryo, endosperm	54	Figure S16
		7.47 (2.57E-2)							
	*Glyma20g29600*	9.26(1.60E-2)	53.66	*AT5G07280*	73.9	*EXS*/*EMS1*	Leucine-rich repeat (LRR) receptor kinase; embryo, endosperm	55	Figure S17
	*Glyma04g37760; Glyma05g38540*	6.88–10.96	46.24–57.80	*AT5G62000*	70.1–72.1	*ARF2*	auxin-responsive element binding transcription factor; integument before pollination, embryo after pollination	56	Figure S18
	*Glyma06g17320; Glyma08g01100*	(1.07E-2–3.05E-2)							
	*Glyma19g36100*	8.42(1.98E-2)	51.28	*AT2G37260*	61.8	*TTG2*	WRKY transcription factor; Integument	57	Figure S19
	*Glyma05g22970; Glyma17g17010*	6.33(3.61E-2)	44.16;51.39	*AT4G37750*	60.5; 61.6	*ANT*	AP2-like transcription factor; Integument before pollination, embryo after pollination	58	Figure S20
		8.46 (1.96E-2)							
