# Supplementary material for: Identification of domestication-related loci associated with flowering time and seed size in soybean with the RAD-seq genotyping method
Source: Sci Rep. 2015 Mar 23;5:9350. doi: 10.1038/srep09350 (PMC4369735; doi:10.1038/srep09350)
Supplement: Supplementary Information [file srep09350-s1.pdf]

Identification of domestication-related loci associated with flowering time and seed size in soybean with the RAD-seq genotyping method

Ling Zhou[1,☯], Shi-Bo Wang[1,☯], Jianbo Jian[2,☯], Qing-Chun Geng[1], Jia Wen[1], Qijian Song[3], Zhenzhen Wu[2], Guang-Jun Li[4], Yu-Qin Liu[5], Jim M. Dunwell[6], Jin Zhang[1], Jian-Ying Feng[1], Yuan Niu[1], Li Zhang[1], Wen-Long Ren[1] & Yuan-Ming Zhang[1,7,*]

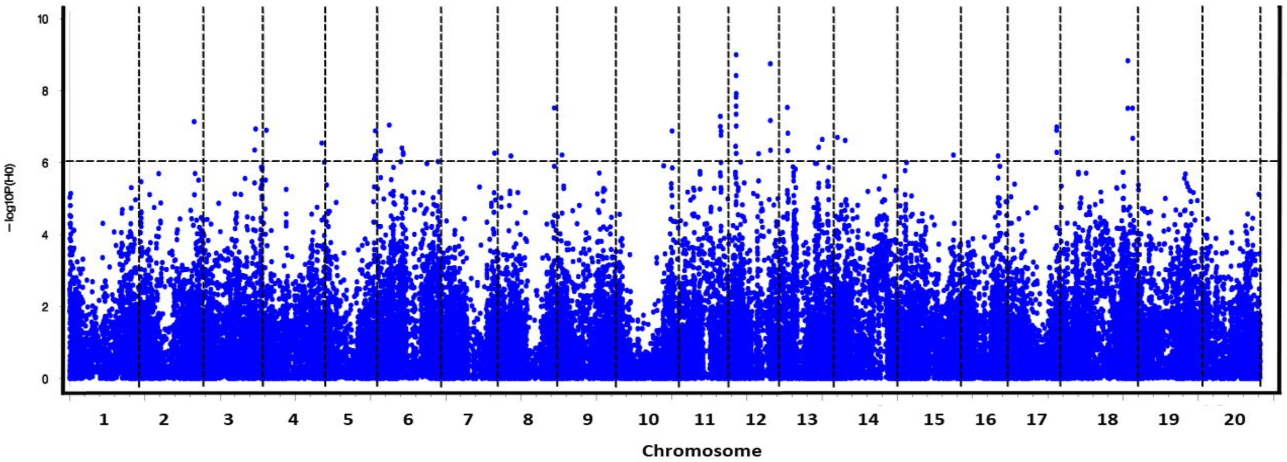

First flowering time in 2010

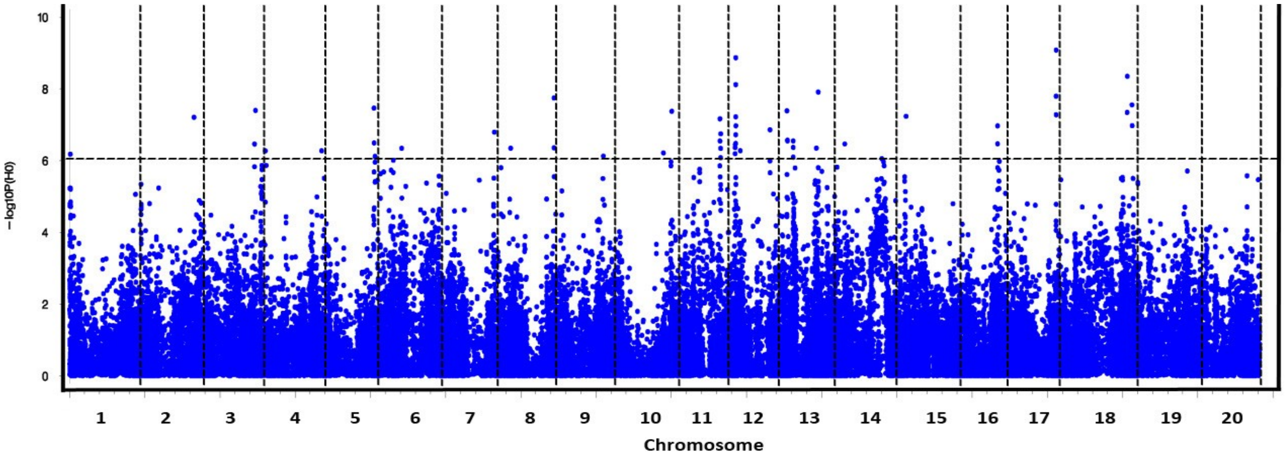

First flowering time in 2011

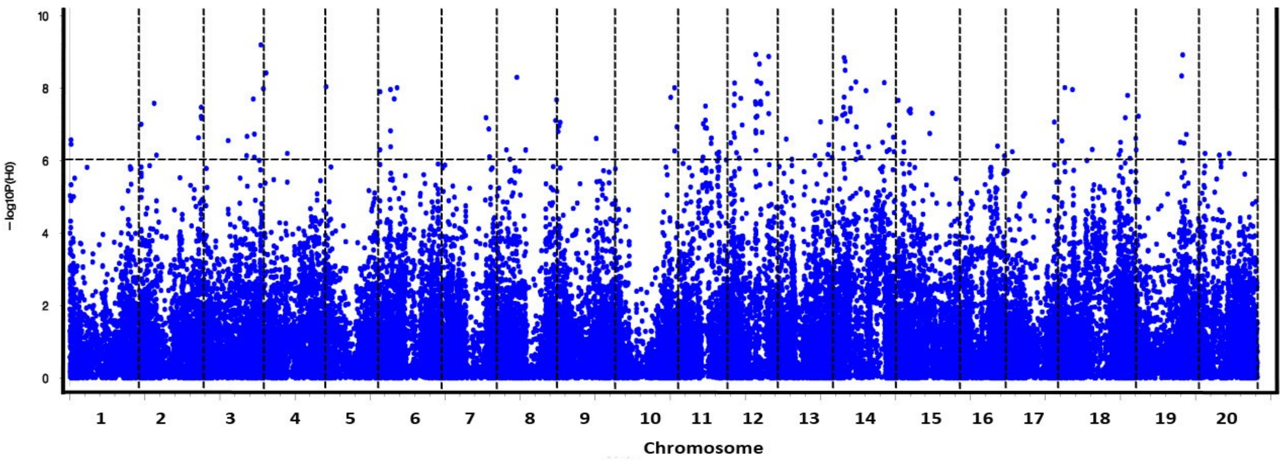

First flowering time in 2012

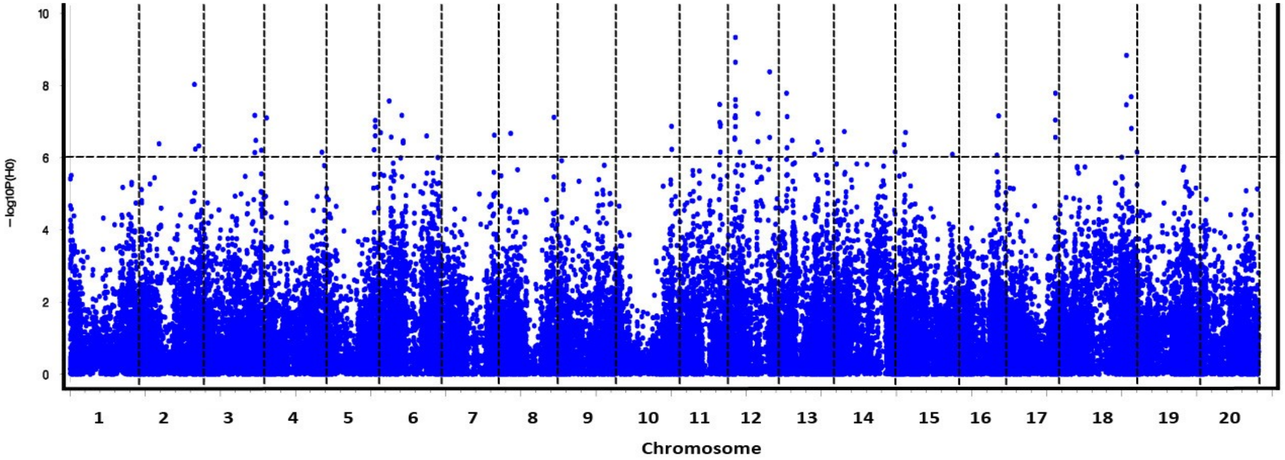

Full flowering time in 2010

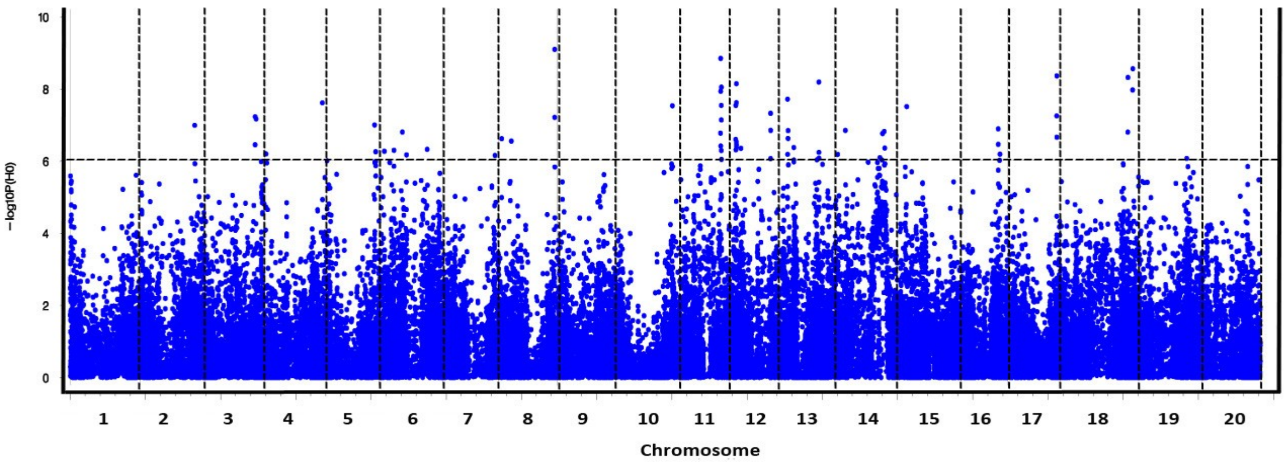

Full flowering time in 2011

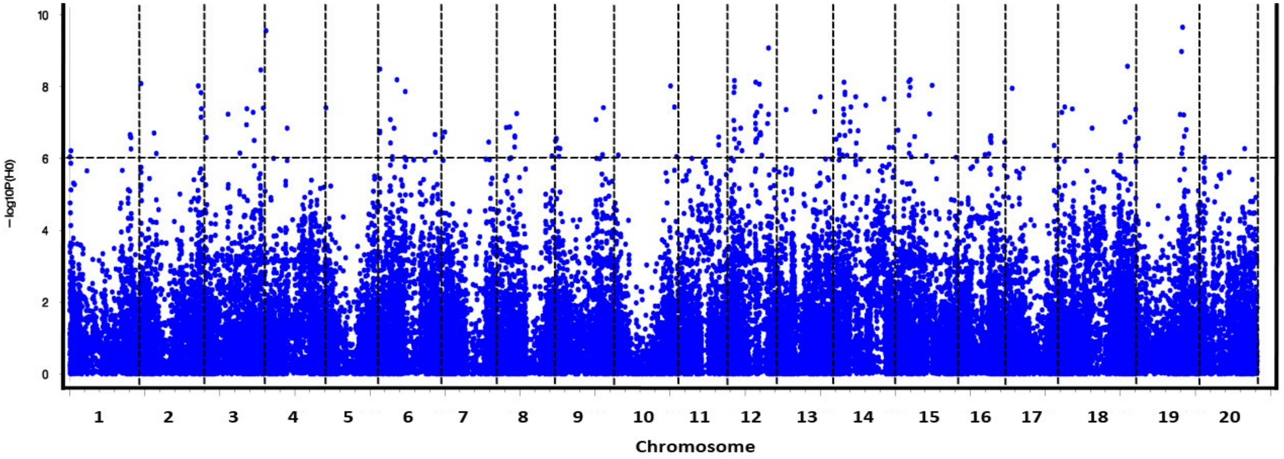

Full flowering time in 2012

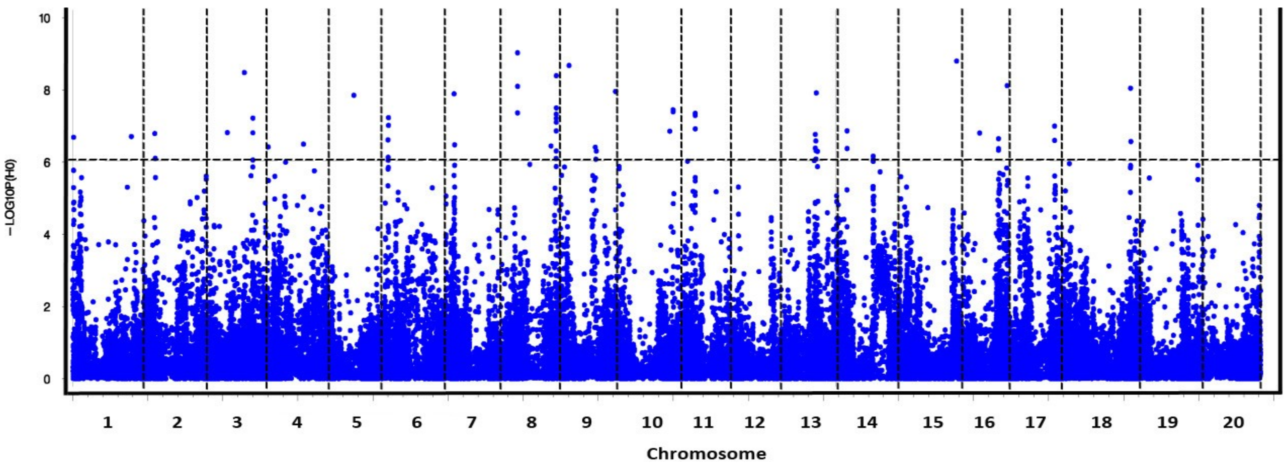

Seed length in 2008

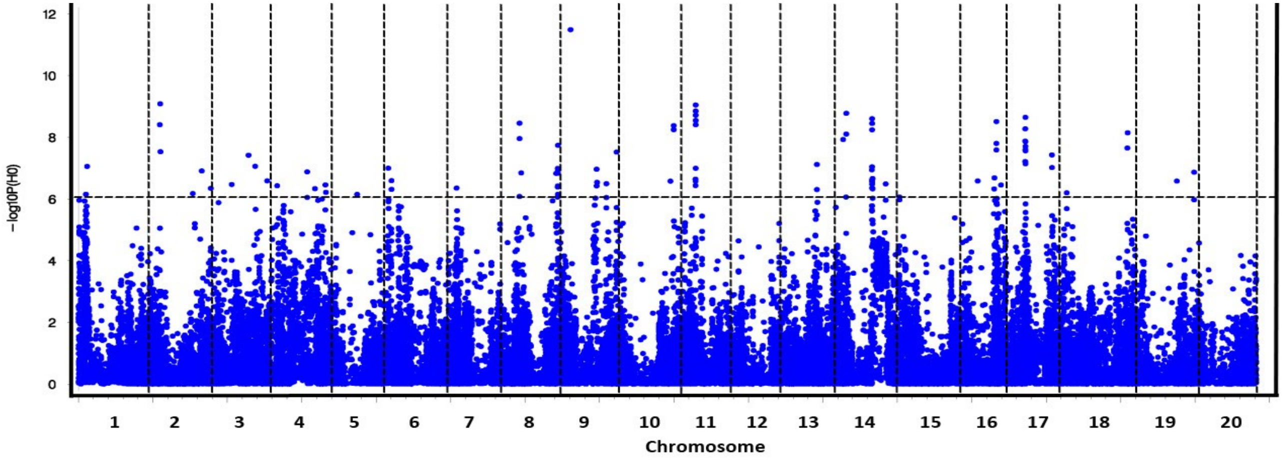

Seed length in 2009

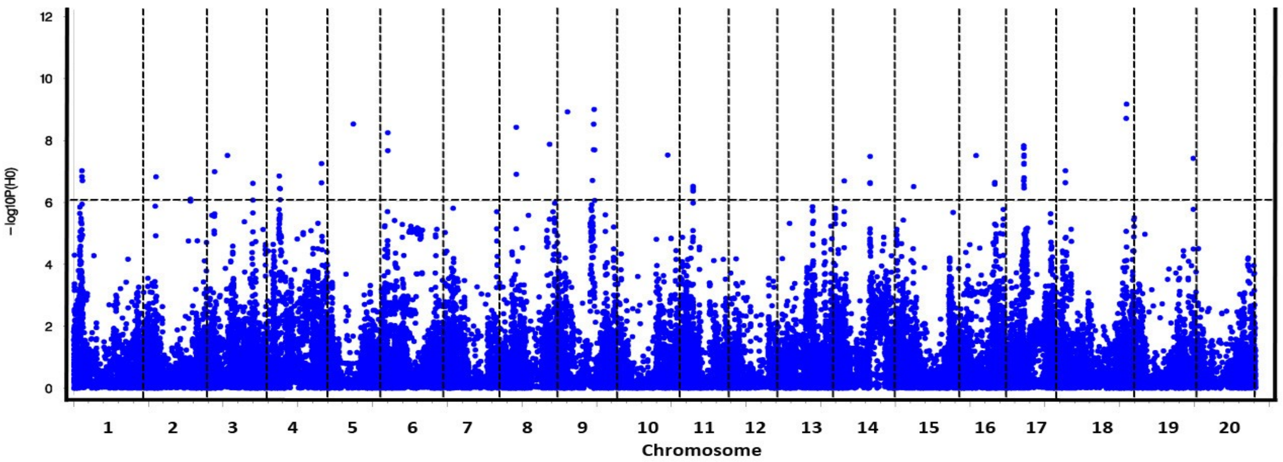

Seed length in 2010

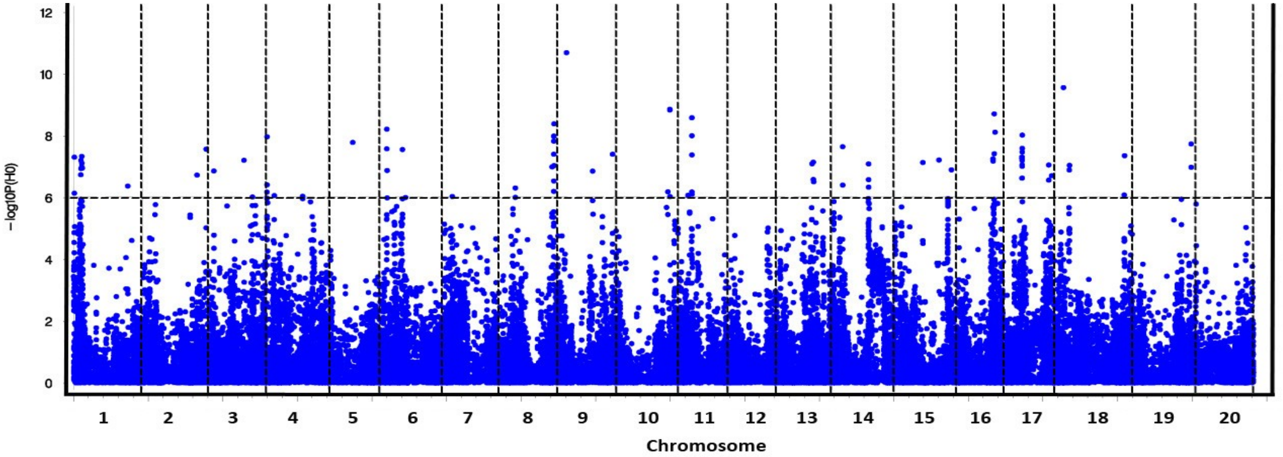

Seed length in 2011

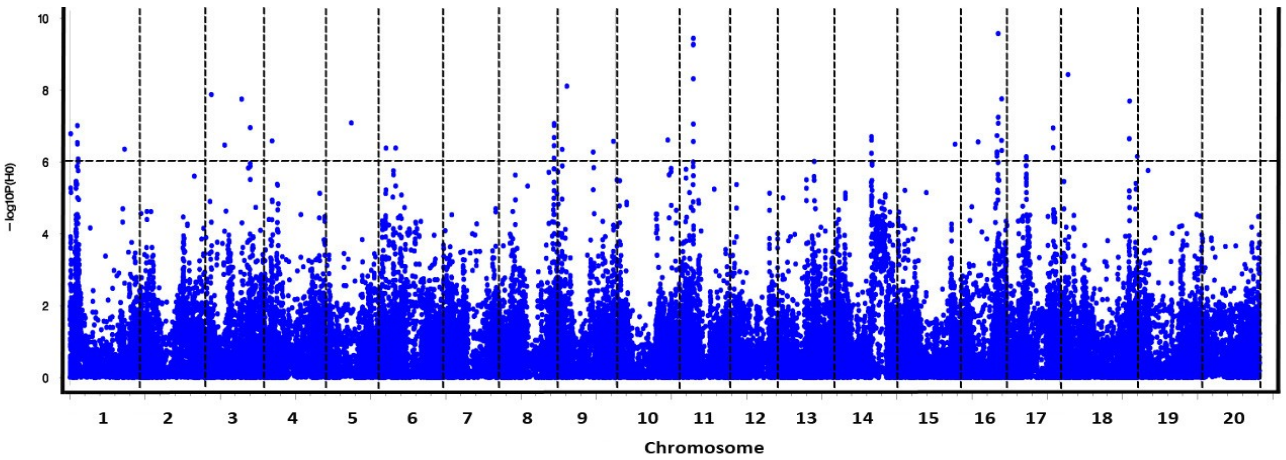

Seed length in 2012

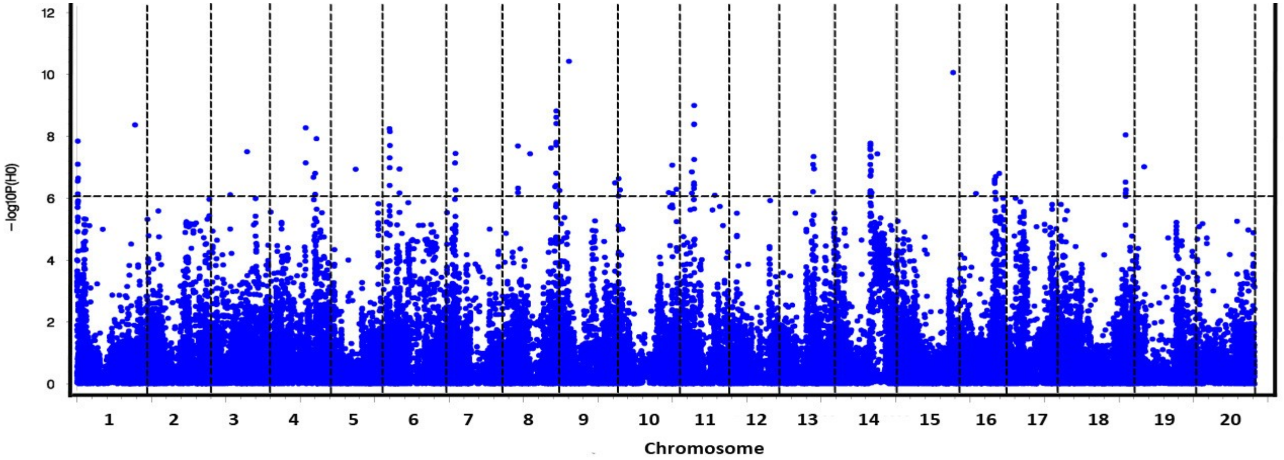

Seed width in 2008

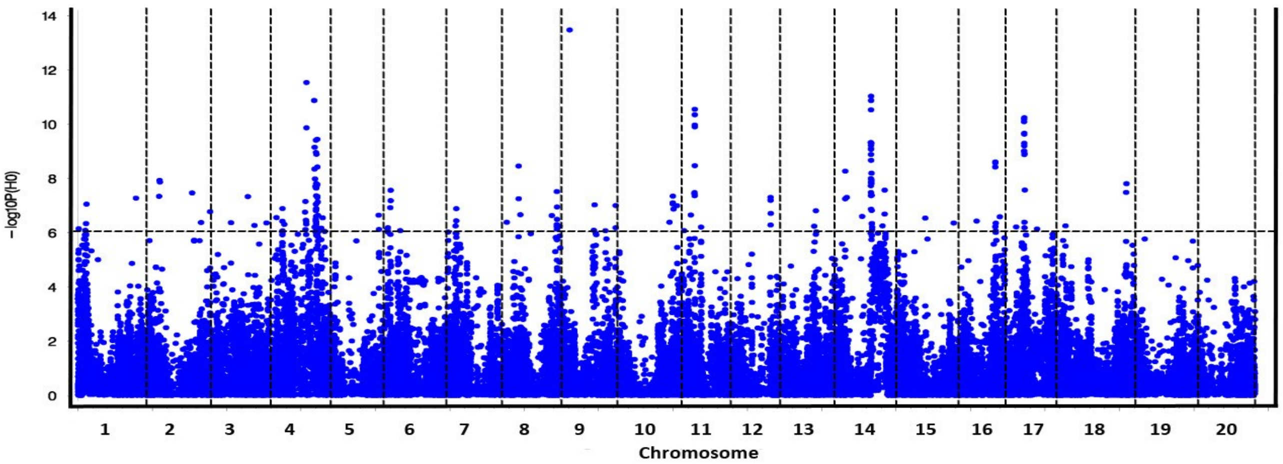

Seed width in 2009

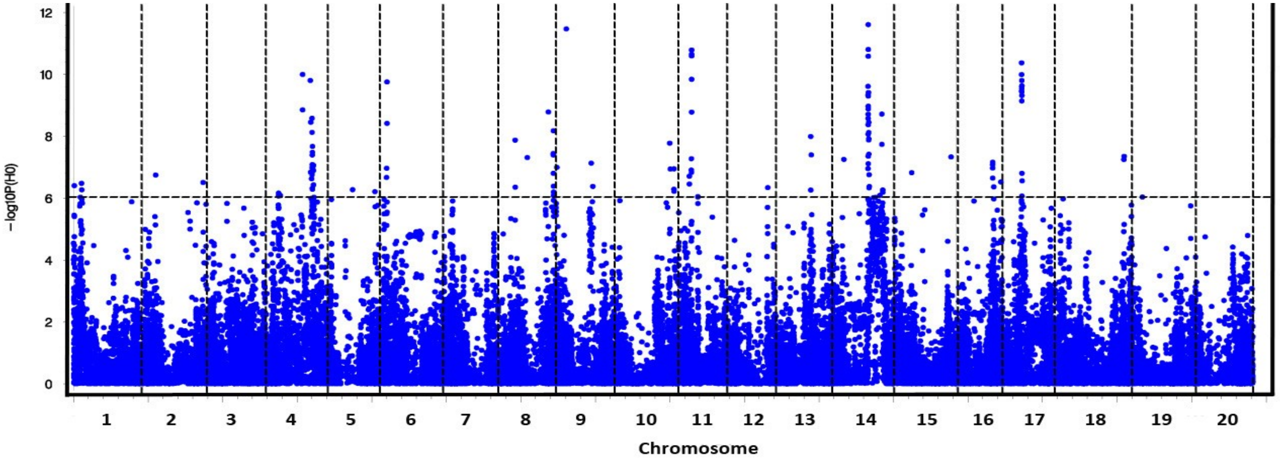

Seed width in 2010

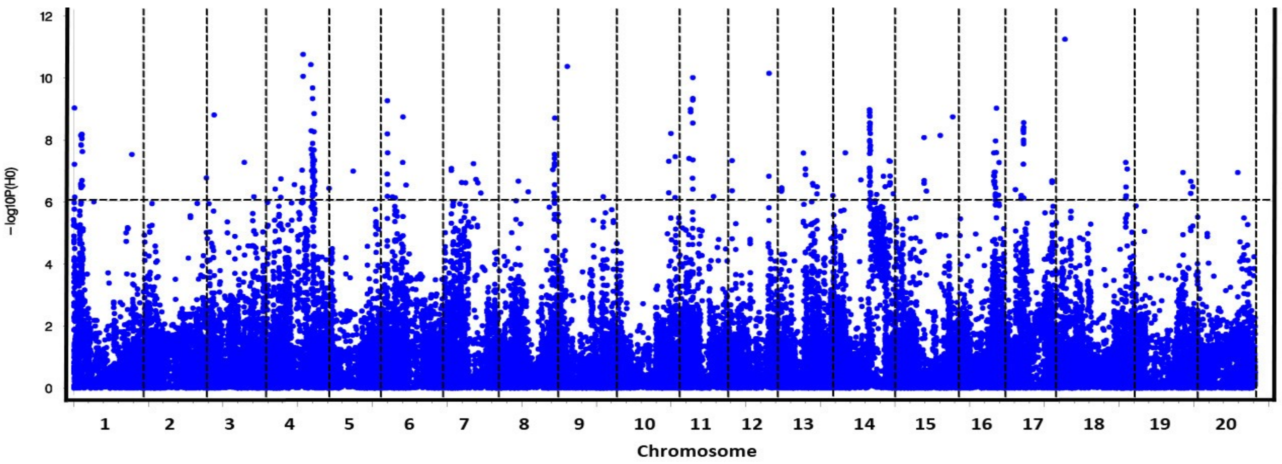

Seed width in 2011

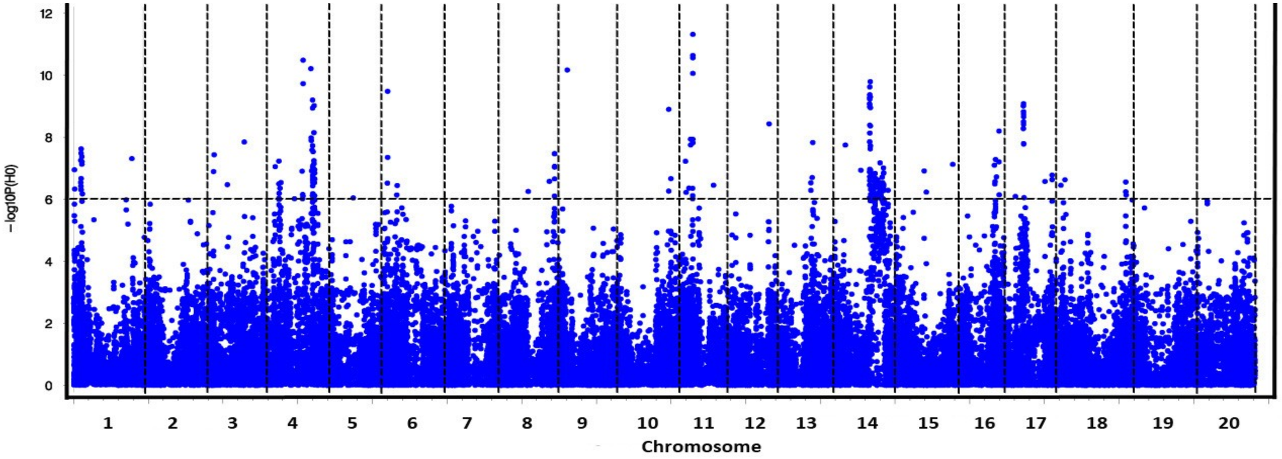

Seed width in 2012

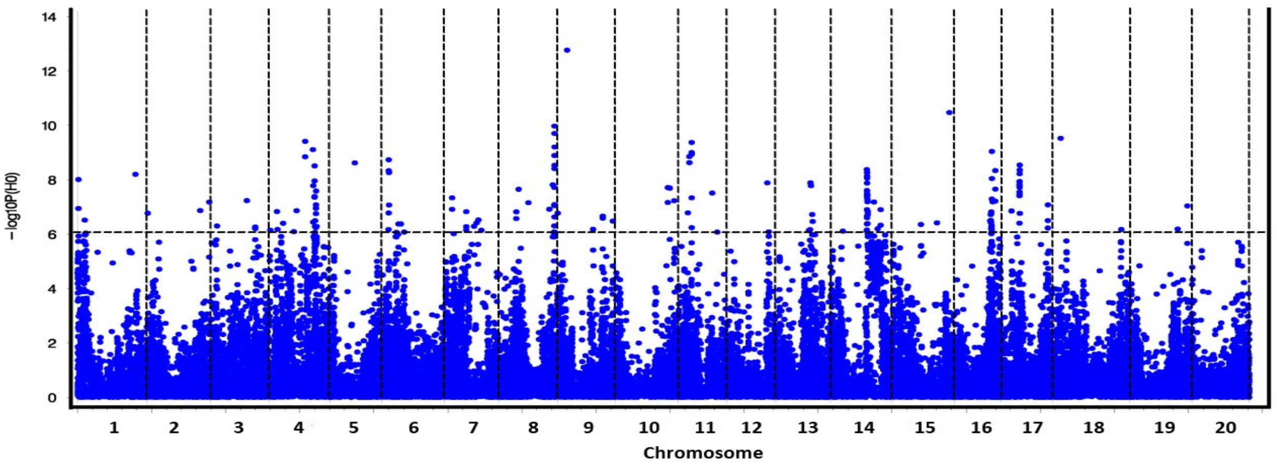

100-seed weight in 2011

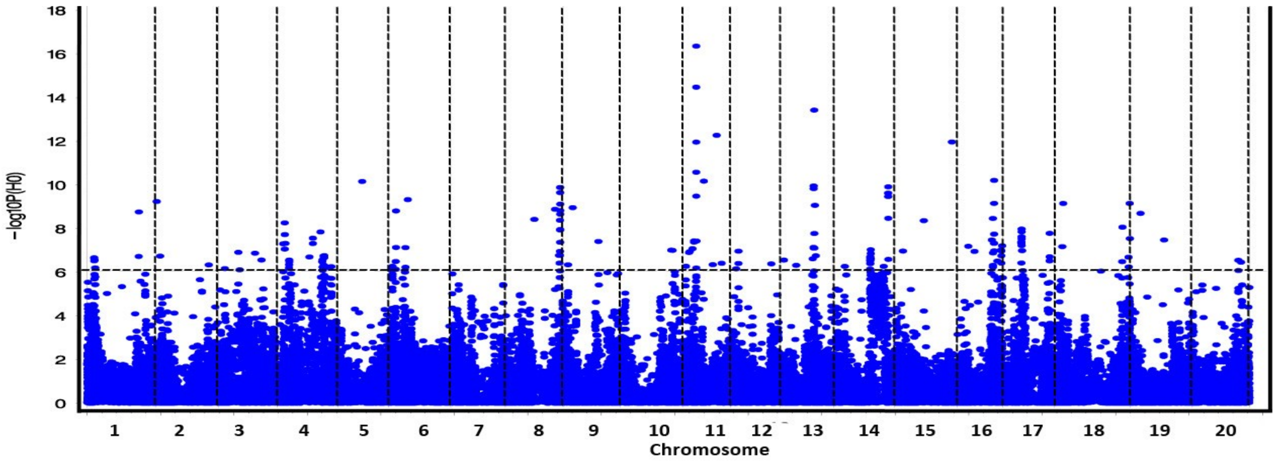

100-seed weight in 2012

Figure S1. **Genome-wide association studies of first and full flowering time, seed length, seed width and 100-seed weight in soybean during**

2008-2012 based on 55,052 SNPs. The genomic positions of SNPs on chromosomes 1 to 20 were 7166-55874947, 2395-51643854, 45230-47773436, 15992-49228296, 9016-41933701, 1142-50641309, 37534-44659030, 3583-46944564, 25976-46841908, 24539-50962464, 30293-39163227, 13701-40093893, 10047-44402574, 13165-49710404, 8319-50879005, 14132-37370388, 47058-41905331, 426-62303776, 9282-50584403 and 18252-46703751 (bp), respectively.

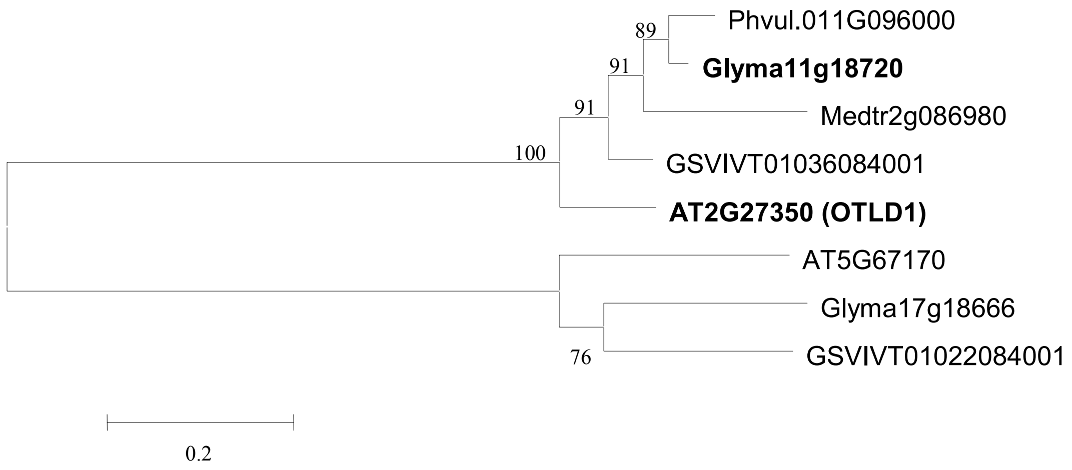

Figure S2. Phylogenetic analysis for the gene family of *Glyma11g18720*. The tree, and all those given in the figures below, was constructed using the neighbor joining method implemented in MEGA 5.0.

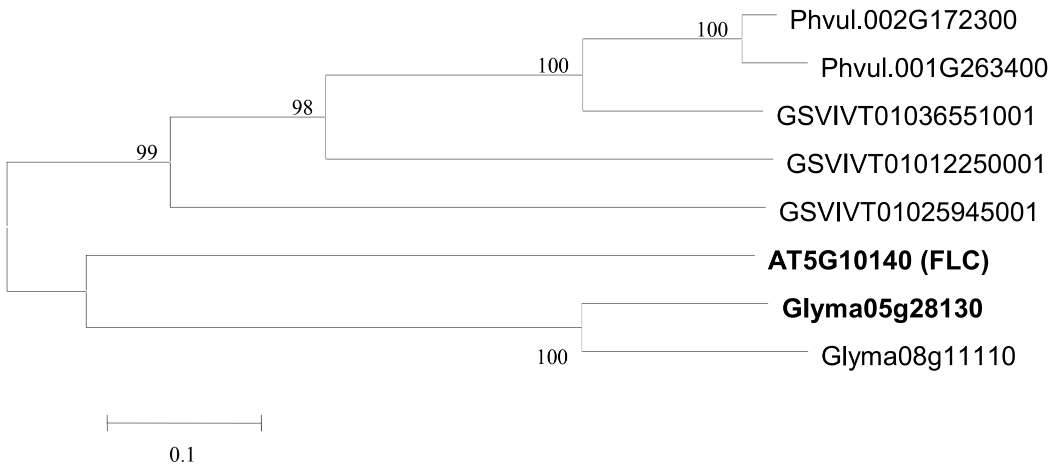

Figure S3. Phylogenetic analysis for the gene family of *Glyma05g28130*.

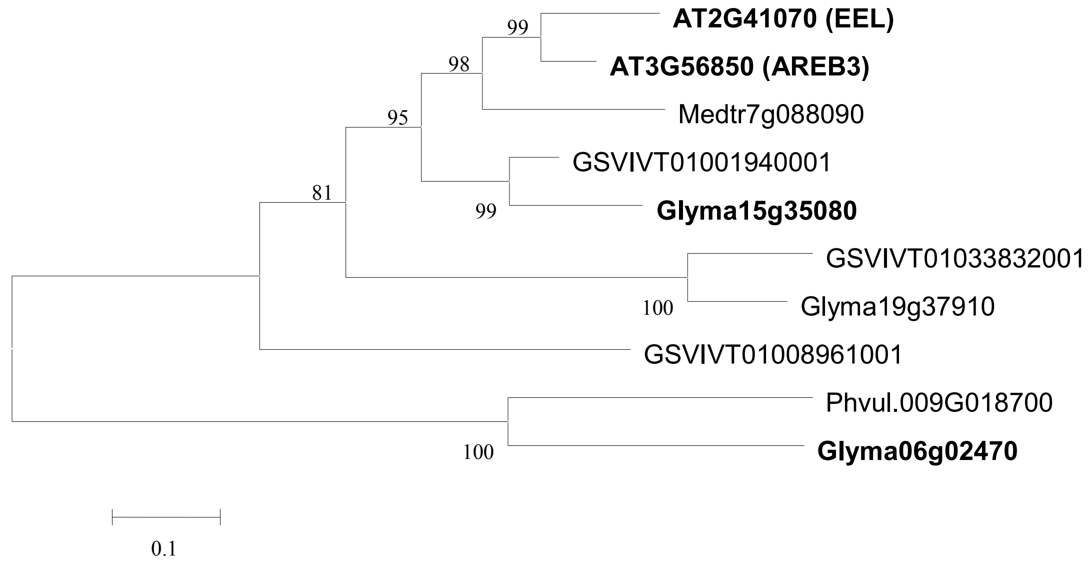

Figure S4. Phylogenetic analysis of the gene family of *Glyma15g35080* and *Glyma06g02470*.

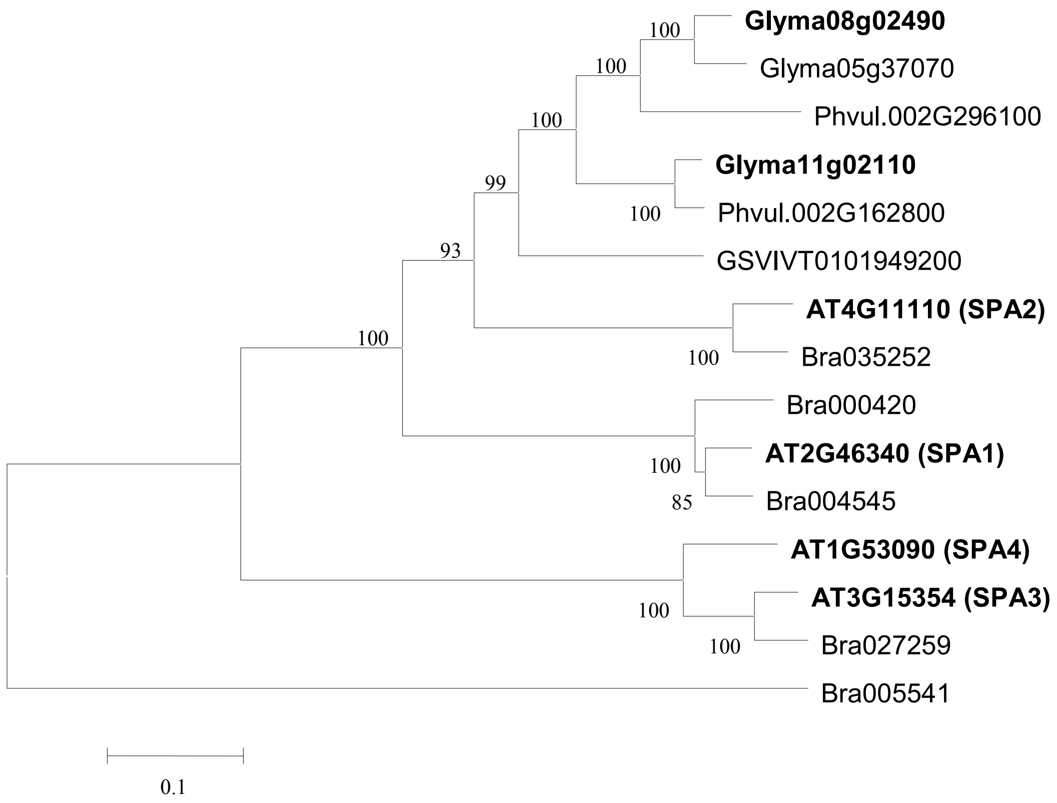

Figure S5. Phylogenetic analysis for the gene family of *Glyma08g02490* and *Glyma11g02110*.

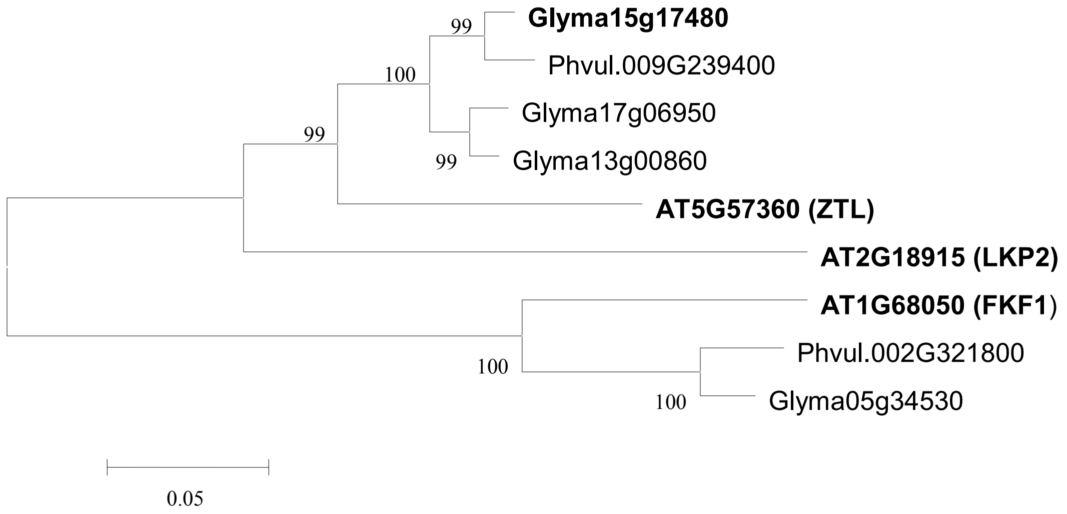

Figure S6. Phylogenetic analysis for the gene family of *Glyma15g17480*.

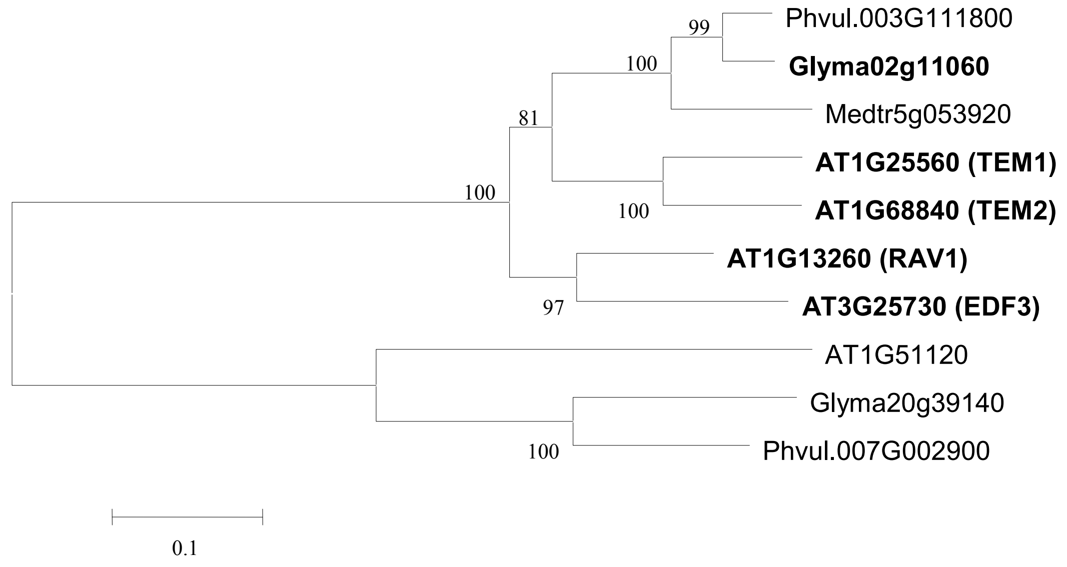

Figure S7. Phylogenetic analysis for the gene family of *Glyma02g11060*.

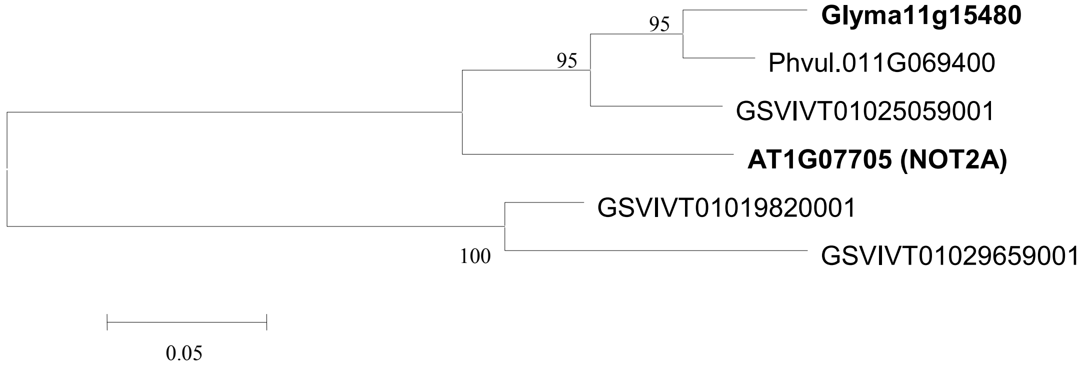

Figure S8. Phylogenetic analysis for the gene family of *Glyma11g15480*.

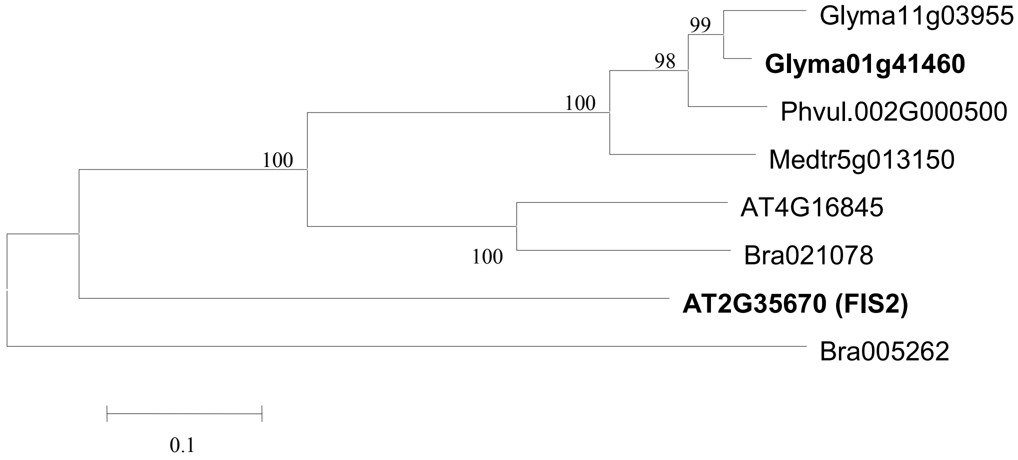

Figure S9. Phylogenetic analysis for the gene family of *Glyma01g41460*.

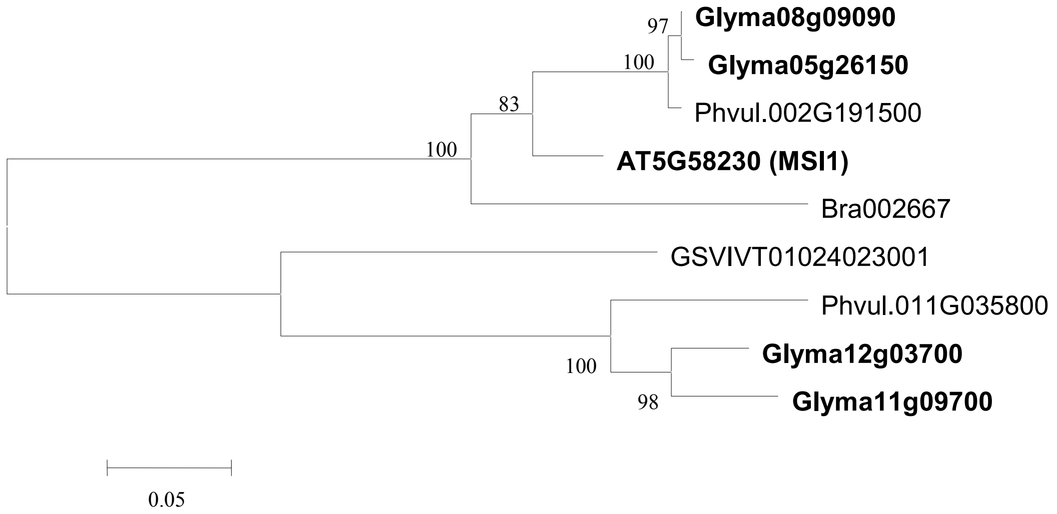

Figure S10. Phylogenetic analysis for the gene family of *Glyma08G09090*, *Glyma05g26150*, *Glyma11g09700* and *Glyma12g3700*.

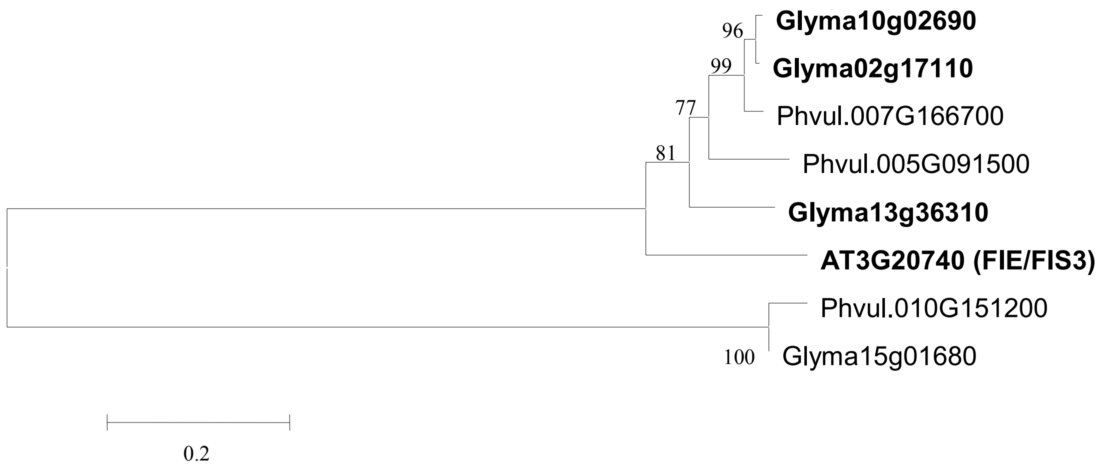

Figure S11. Phylogenetic analysis for the gene family of *Glyma02g17110*, *Glyma10g02690* and *Glyma13g36310*.

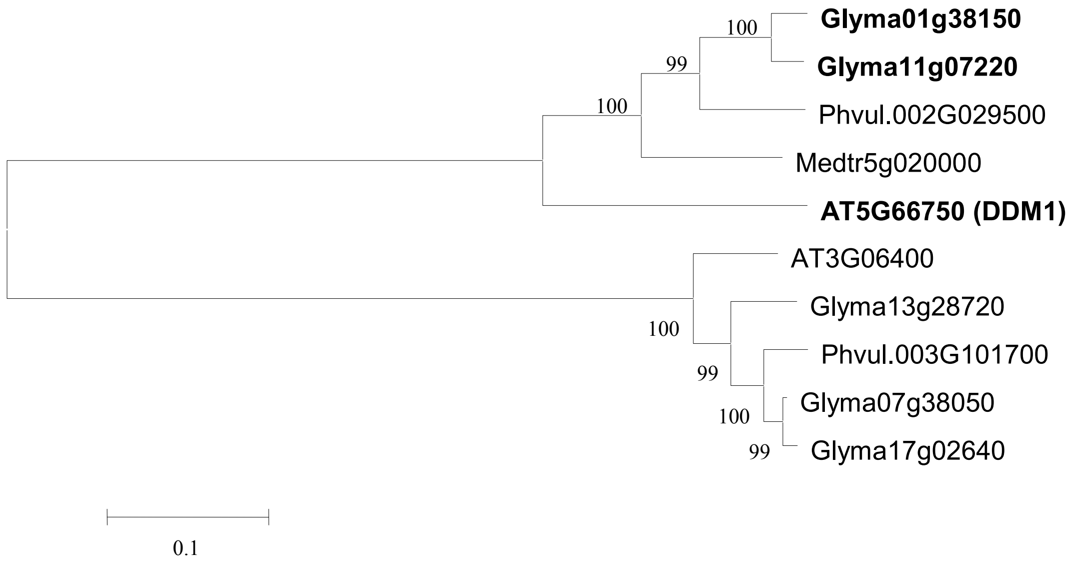

Figure S12. Phylogenetic analysis for the gene family of *Glyma01g38150* and *Glyma11g07220*.

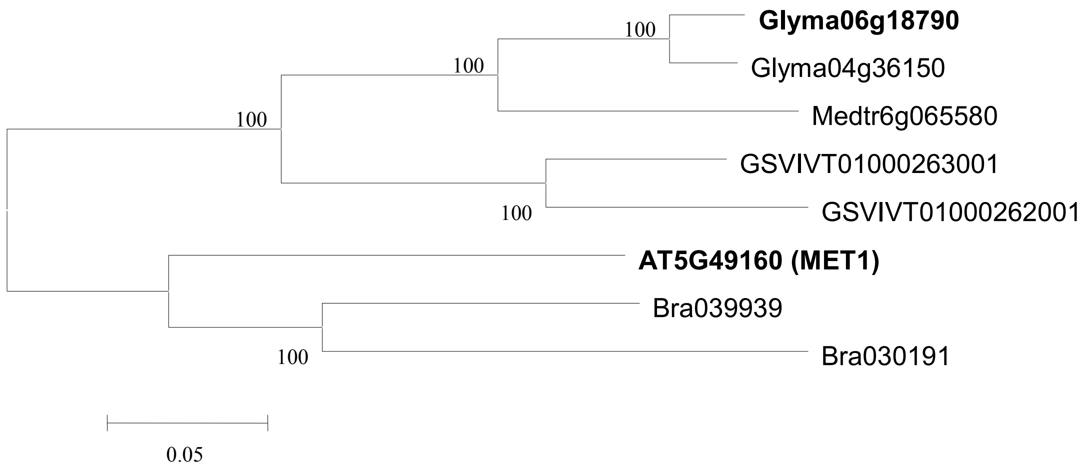

Figure S13. Phylogenetic analysis for the gene family of *Glyma06g18790*.

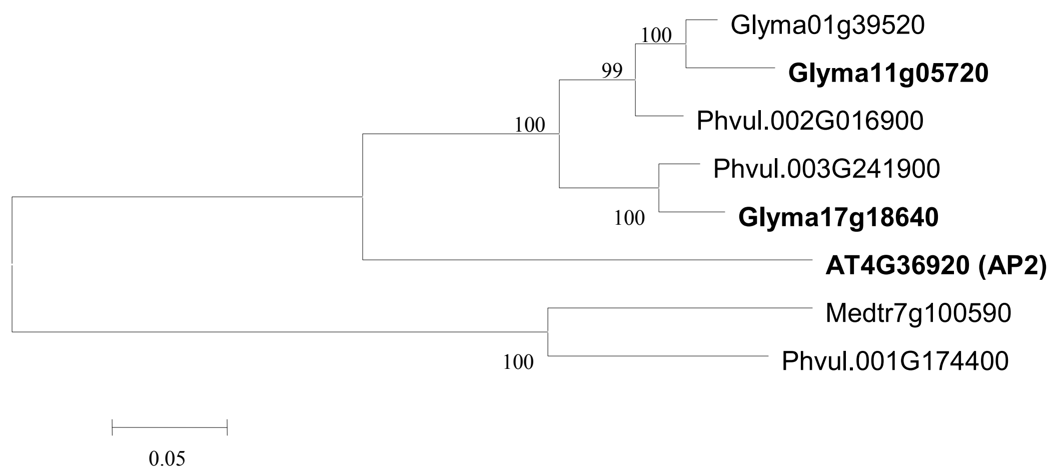

Figure S14. Phylogenetic analysis of the gene family of *Glyma11g05720* and *Glyma17g18640*.

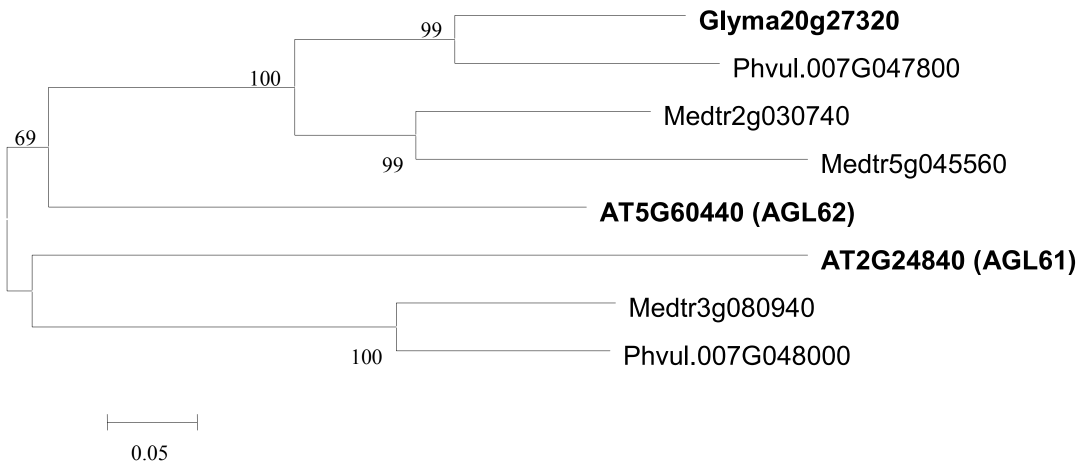

Figure S15. Phylogenetic analysis for the gene family of *Glyma20g27320*.

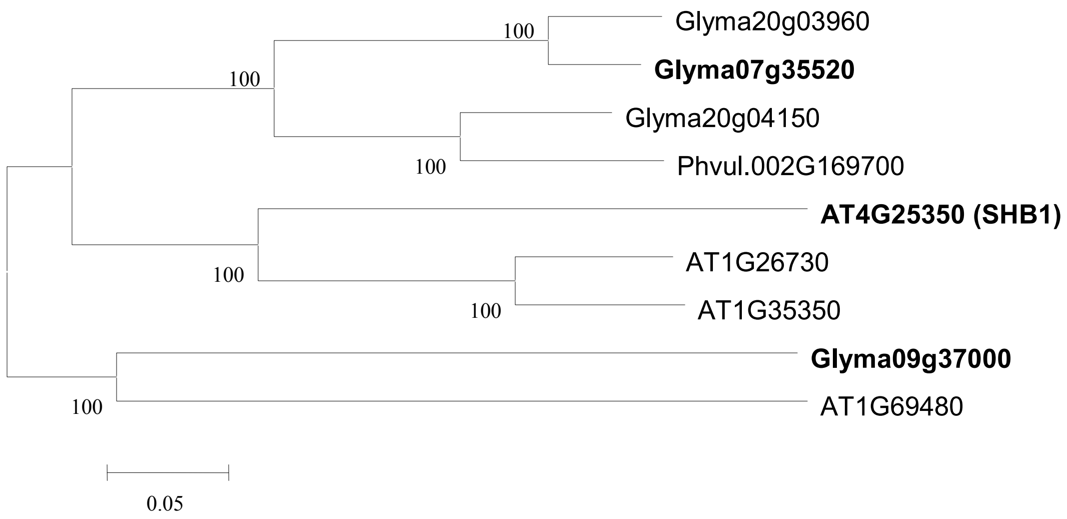

Figure 16. Phylogenetic analysis for the gene family of *Glyma07g35520* and *Glyma09g37000*.

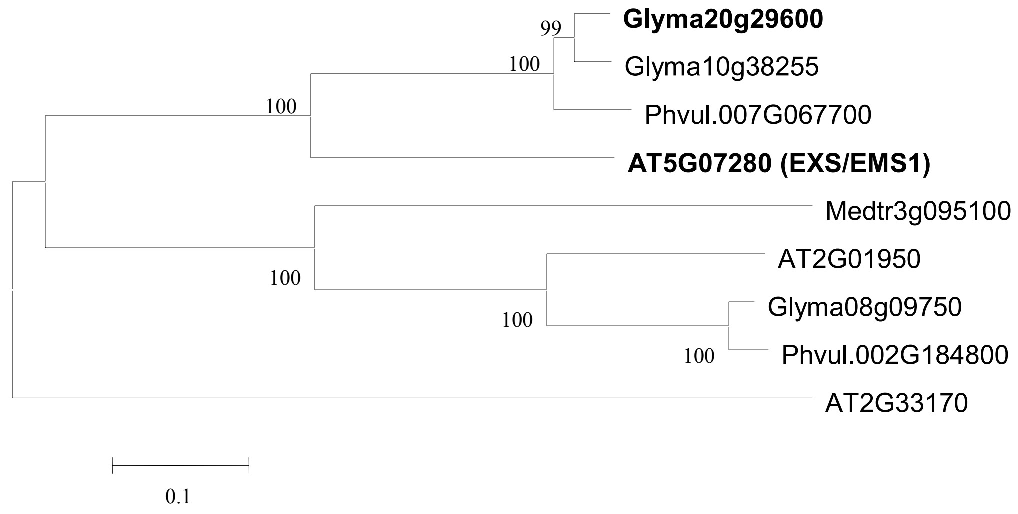

Figure S17. Phylogenetic analysis for the gene family of *Glyma20g29600*.

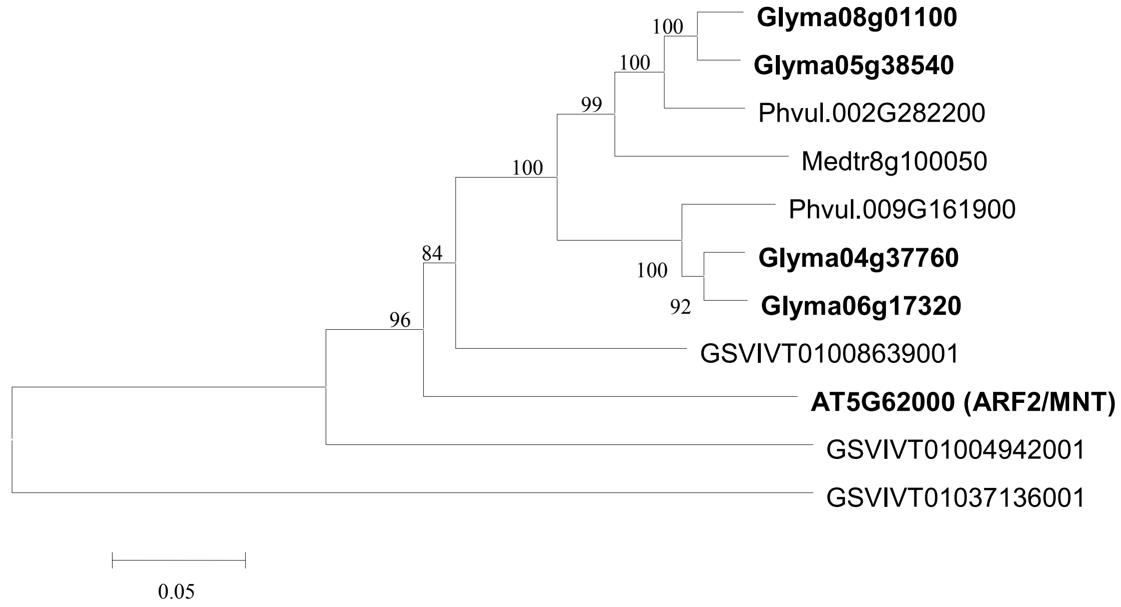

Figure S18. Phylogenetic analysis for the gene family of *Glyma04g37760*, *Glyma05g38540*, *Glyma06g17320* and *Glyma08g01100*.

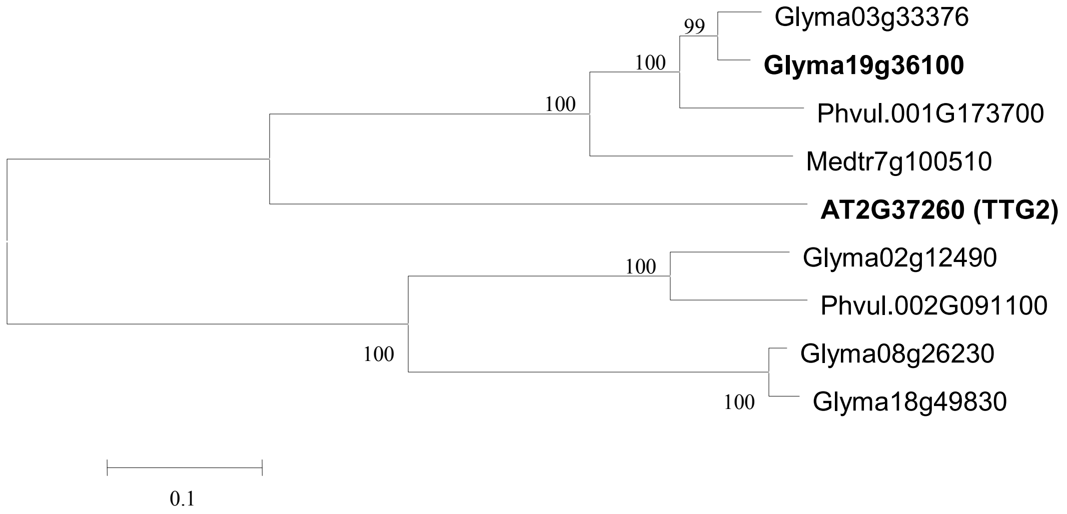

Figure S19. Phylogenetic analysis for the gene family of *Glyma19g36100*.

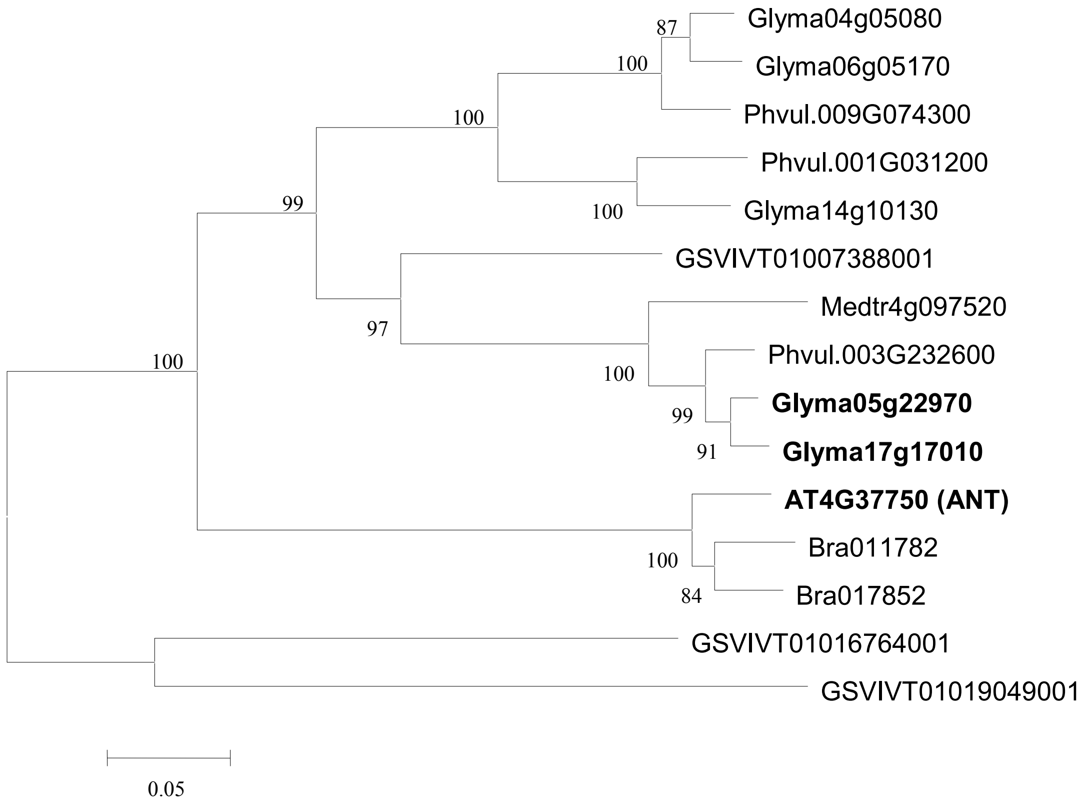

Figure S20. Phylogenetic analysis for the gene family of *Glyma05g22970* and *Glyma17g17010*.

Table S1. Domestication-related loci in soybean genome determined by χ^2 test of independence, U-test and genetic diversity analysis

Chr.	Position (bp)	χ^2 ($-\log_{10} P$)	U ($-\log_{10} P$)	F_{ST}	Chr.	Position (bp)	χ^2 ($-\log_{10} P$)	U ($-\log_{10} P$)	F_{ST}	Chr.	Position (bp)	χ^2 ($-\log_{10} P$)	U ($-\log_{10} P$)	F_{ST}
1	1639236	19.15(6.33)	-4.18(4.53)	0.7878	11	15299054	30.64(6.65)	-2.74(2.21)	0.5884	15	39607166	32.10(6.97)	-3.89(3.40)	0.6496
1	2773896	29.71(6.45)	-4.04(4.27)	0.5819	11	15311868	33.87(7.36)	-4.28(4.73)	0.76	15	41394266	42.58(9.25)	-2.95(2.50)	0.6405
1	2829130	31.54(6.85)	-4.19(6.05)	0.8063	11	15352584	44.21(9.60)	-2.37(1.75)	0.566	15	41406180	46.09(10.00)	-3.87(3.97)	0.7532
6	8118481	29.77(6.46)	-5.93(8.53)	0.8518	11	15362036	45.45(9.87)	-2.43(1.82)	0.5656	15	41495011	31.63(6.87)	-3.61(3.51)	0.6906
6	12953089	31.77(6.90)	-5.81(8.22)	0.8665	11	15363309	49.74(10.80)	-2.22(1.58)	0.5964	15	41547491	29.41(6.39)	-3.47(3.29)	0.5352
6	15695551	31.93(6.94)	-3.58(6.44)	0.8506	11	15385477	48.87(10.61)	-2.88(2.40)	0.6045	15	41547525	32.84(7.13)	-3.56(3.42)	0.7176
6	15896953	32.05(6.96)	-3.58(3.47)	0.6664	11	15385495	48.84(10.61)	-2.88(2.40)	0.6045	15	41698739	32.90(7.15)	-4.30(4.77)	0.7365
7	10165145	29.53(6.41)	-5.05(6.36)	0.823	11	15395305	47.79(10.38)	-2.36(1.74)	0.5285	15	41698758	32.90(7.15)	-4.30(4.77)	0.7365
9	46354191	36.36(7.90)	-3.82(3.88)	0.6743	12	5895633	37.23(8.08)	-3.73(3.71)	0.7344	15	41729760	31.53(6.85)	-3.43(3.23)	0.6878
9	46504193	37.20(8.08)	-3.24(2.93)	0.6329	12	5895655	38.04(8.26)	-4.30(4.78)	0.783	15	41770224	29.42(6.39)	-3.10(2.72)	0.6303
11	10957940	30.60(6.64)	-2.36(1.74)	0.489	12	5895785	38.74(8.41)	-4.19(4.55)	0.7938	16	5682870	29.37(6.38)	-3.80(3.83)	0.646
11	11111516	38.40(8.34)	-2.53(1.95)	0.5069	12	5957099	30.47(6.62)	-2.78(2.27)	0.6017	16	30207175	30.67(6.66)	-3.47(3.28)	0.5348
11	15142904	32.74(7.11)	-4.18(4.54)	0.7219	12	33215509	30.36(6.59)	-7.44(13.01)	0.9297	16	30210113	30.90(6.71)	-3.74(3.74)	0.6148
11	15147848	31.53(6.84)	-4.19(4.55)	0.7355	13	7661137	30.18(6.55)	-3.41(3.19)	0.6441	17	2690293	30.44(6.61)	-3.41(3.19)	0.6305
11	15170736	32.34(7.02)	-3.52(3.37)	0.6606	13	22953389	31.28(6.79)	-2.15(1.51)	0.4702	17	2690302	30.44(6.61)	-3.41(3.19)	0.6305
11	15252435	39.57(6.42)	-4.52(5.22)	0.6178	15	34325359	33.85(7.35)	-2.03(1.38)	0.4995	17	3193537	35.17(7.64)	-2.72(2.18)	0.5653

Table S2. SNPs and allele frequencies of SNPs in the coding (bold) and non-coding regions of eight candidate genes of flowering time or seed size in wild and cultivated soybeans

Gene	Position (bp)	SNPs and allele frequency in this study				SNPs & allele frequency in Chung et al. (2014)				SNPs and allele frequency in Lam et al. (2010)							
		Position	REF/ALT	Cultivated soybean (272)	Wild(14)	Position	REF/ALT	Cultivated soybean(10)	Wild(6)	Position	REF/ALT	Cultivated soybean (17)	Wild(14)	Position	REF/ALT	Cultivated soybean (17)	Wild(14)
Glyma11g15300	10946180-10950711					10946907	C/A	2A/8C	3A/3C	10946191	A/G	1G/16A	0G/14A	10949117	G/A	3A/11G	0A/13G
										10946907	C/A	2A/11C	1A/10C	10949523	C/A	4A/12C	0A/14C
										10947023	T/C	2C/11T	0C/14T	**10950148**	**G/A**	**4A/11G**	**0A/14G**
										**10947579**	**T/A**	**4A/12T**	**1A/13T**	**10950185**	**A/G**	**3G/12A**	**0G/14A**
										**10947751**	**G/T**	**2T/12C**	**0T/14C**	10950511	G/C	10C/5G	0C/14G
										**10948541**	**C/T**	**3T/13C**	**0T/14C**	10950711	T/A	2A/13T	0A/14T
Glyma11g15480	11108543-11116620	**11111516**	**C/A**	**181A/67C**	**11A/2C**	11110941	C/T	1T/9C	5T/1C	11108571	C/T	9T/6C	1T/12C	11112550	C/A	3A/12C	0A/14C
						**11111516**	**C/A**	**2A/8C**	**5A/1C**	11108675	G/T	2T/15G	0T/14G	11112715	C/A	2A/14C	0A/14C
						11113542	T/G	1G/9T	5G/1T	11108709	G/C	3C/13G	0C/14G	11112897	T/C	2C/12T	0C/14T
						11113572	T/G	2G/8T	5G/1T	11108728	G/T	2T/14G	0T/14G	11113208	A/T	2T/1A	0T/11A
						11113586	A/G	1G/9A	5G/1A	11108817	T/A	6A/8T	1A/13T	11113209	G/A	2A/1G	0A/11G
						**11113620**	**T/C**	**2C/8T**	**5C/1T**	11108862	G/A	9A/6G	0A/13G	11113218	A/T	0T/3A	1T/10A
						11114706	G/A	10A/0G	6A/0G	11108949	C/T	2T/14C	0T/13C	11113219	T/A	1A/2T	1A/10T
						11115052	T/C	2C/8G	5C/1G	11109102	T/G	8G/6T	1G/13T	11113332	T/C	10C/3T	1C/11T

11109107	T/C	8C/6T	1C/12T	11113369	A/T	2T/2A	0T/12A
11109361	G/T	2T/12T	1T/13G	11113398	G/C	3C/2G	1C/12G
11109519	G/T	8T/7G	1T/13G	11113542	T/G	5G/10T	0G/14T
11109539	G/A	0A/15G	2A/12G	11113572	T/G	14G/2T	1G/13T
11109731	T/A	8A/7T	1A/13T	11113586	A/G	1G/14A	0G/14A
11109783	G/T	7T/9G	1T/13G	**11113620**	**T/C**	**13C/2T**	**1C/13T**
11110941	C/T	4T/12C	0T/14C	**11113857**	**C/A**	**12A/3C**	**1A/13C**
11111326	A/T	2T/11A	0T/14A	11114684	C/T	3T/12C	1T/12C
11111327	T/A	2A/10T	0A/14T	11114688	C/T	14T/3C	1T/12C
11111328	A/T	2T/8A	0T/14A	11114840	G/A	2A/14G	0A/13G
11111334	G/T	2T/8G	0T/13G	11115052	T/C	13C/3T	1C/12T
**11111483**	**T/G**	**2G/13T**	**0G/14T**	11115349	C/A	2A/13C	0A/14C
**11111516**	**C/A**	**13A/3C**	**1A/13C**	11115356	A/T	14T/3A	1T/13A
**11111536**	**T/C**	**2C/13T**	**0C/14T**	11115558	T/G	3G/12T	0G/14T
11111744	A/G	3G/14A	1G/13A	11115627	G/A	3A/12G	0A/14G
11111964	A/G	13G/3A	1G/13A	11115913	C/T	2T/13C	1T/13C
11112199	C/T	14T/3C	1T/13C	11116034	A/T	13T/3A	1T/12A
11112262	A/T	13T/2A	1T/13A	11116042	C/G	14G/3C	1G/12C

										11112353	A/G	13G/3A	1G/13A	11116301	A/C	2C/14A	0C/14A
										11112377	C/T	13T/2C	1T/13C	11116464	G/A	3A/12G	0A/14G
										11112424	C/T	9T/3C	1T/13C	11116542	T/G	3G/13T	1G/13T
										11112483	T/C	2C/13T	1C/13T	11116618	C/T	6T/9C	1T/13C
Glyma11g18720	15353228-15363245	**15362036**	**A/C**	**187C/63A**	**9C/3A**	15353360	T/C	0C/10T	2C/4T	15353798	C/G	11G/3C	1G/13C	15359850	A/C	14C/1A	1C/13A
						**15354919**	**G/A**	**2A/8G**	**6A/0G**	15354449	G/A	2A/14G	0A/14G	15361202	T/C	2C/7T	0C/12T
						**15355349**	**C/T**	**0T/10C**	**2T/4C**	15354919	G/A	14A/1G	1A/13G	15361208	C/T	1T/9C	0T/13C
						15355799	A/G	2G/9A	6G/0A	15355205	T/G	11G/1T	1G/13T	15361303	G/A	11A/2G	1A/12G
						15356793	T/A	4A/6T	6A/0T	15355387	G/A	11A/2G	1A/13G	15361852	T/G	13G/2T	1G/13T
						15357681	C/T	2T/8C	6T/0C	15355458	A/G	10G/1A	1G/13A	15361857	T/G	2G/14T	0G/14T
						15359329	G/A	0A/10G	2A/4G	15355497	A/G	10G/2A	1G/13A	**15361982**	**G/A**	**12A/2G**	**1A/13G**
						15359850	A/C	2C/8A	6C/0A	15355799	A/G	14G/1A	1G/13A	**15362036**	**A/C**	**12C/1A**	**1C/12A**
										15355854	G/T	15T/1G	1T/13G	**15362320**	**C/A**	**0A/16C**	**1A/13C**
										15356140	G/A	13A/3G	1A/12G	**15362396**	**T/A**	**11A/1T**	**1A/13T**
										15356793	T/A	17A/0T	4A/10T	**15362486**	**C/A**	**1A/15C**	**0A/14C**
										15358607	A/T	7T/3A	0T/12A	15362861	T/A	4A/7T	0A/10T
										15358630	A/T	4T/1A	1T/12A	15363214	C/T	6T/3C	1T/13C
										15358632	C/T	0T/9C	2T/9C	15363219	C/A	1A/5C	0A/14C

						15359102	G/A	12A/3G	1A/13G	15363220	T/G	1G/5T	0G/14T
Glyma12g08150	5874276-5880441	5875950	A/G	10G/0A	6G/0A	5874364	A/G	2G/15A	0G/13A	5876749	T/C	2C/13T	0C/11T
		5876035	A/G	0G/10A	3G/3A	**5874611**	**C/T**	**2T/15C**	**0T/13C**	5877063	T/A	4A/13T	1A/12T
		5876735	G/A	0A/10G	4A/2G	5874750	C/T	2T/14C	0T/14C	5877228	A/G	2G/15A	0G/13A
		5877228	A/G	0G/10A	3G/3A	5874772	C/A	2A/13C	0A/14C	5877710	G/T	1T/14G	1T/13G
		5879514	T/A	0A/10T	2A/4T	5874801	C/T	2T/12C	1T/12C	5877738	A/G	2G/15A	0G/14A
		5879994	A/G	0G/10A	3G/3A	5874805	A/C	3C/13A	0C/13A	5878220	G/A	4A/13G	0A/12G
						5874807	A/G	4G/12A	0G/13A	5878313	A/T	2T/14A	0T/14A
						5874845	T/G	3G/14T	0G/13T	5878370	A/G	2G/14A	0G/12A
						5874848	G/A	3A/14G	0A/13G	5878631	A/G	3G/14A	0G/12A
						**5874949**	**T/A**	**3C/14T**	**0A/14T**	5878635	A/T	3T/14A	0T/12A
						5875359	T/G	3G/14T	0G/13T	5878782	A/G	3G/14A	0G/14A
						5875434	A/C	2C/14A	0C/13A	5878845	T/C	2C/14T	0C13T
						5875894	C/A	2A/13C	0A/14C	5879422	A/G	4G/12A	0G/14A
						5876279	G/A	3A/14G	0A/11G	5879507	A/C	1C/12A	0C/13A
						5876289	A/G	2G/14A	0G/12A	5879514	T/A	6A/9T	0A/13T
						5876483	T/G	4G/11T	0G/13T	5880203	G/A	1A/13G	1A/13G
						5876485	A/T	2T/13A	0T/13A	5880232	A/G	1G/13A	0G/14A

						5876618	C/A	2A/12C	0A/12C	5880336	C/T	4T/11C	1T/13C
						5876735	G/A	4A/10G	1A/10G				
Glyma12g08210	5937860-5944400	**5938534**	**C/G**	**0G/10C**	**2G/4C**	5937882	A/G	9G/5A	3G/10A	5942218	C/G	9G/7C	1G/13C
		5938666	G/C	0C/10G	2C/4G	**5938534**	**C/G**	**4G/13C**	**0G/13C**	5942381	A/T	4T/13A	0T/14A
		5938668	A/C	0C/10A	6C/0A	5938668	A/C	12C/4A	3C/11A	5943256	G/A	12A/5G	3A/11G
						5940121	T/C	10C/7T	1C/13T	5943409	T/A	10A/6T	1A/13T
						5940194	T/A	4A/13T	0A/14T	5943424	T/A	4A/13T	0A/14T
						5941031	A/T	3T/10A	0T/13A	5943775	A/G	4G/13A	0G/14A
						5941085	G/A	2A/15G	1A/13G	5943903	G/A	2A/14G	0A/14G
						5941199	T/C	2C/12T	1C/13T	5943970	A/G	10G/6A	2G/10A
						5941257	T/C	2C/15T	1C/13T	5944242	T/A	2A/12T	0A/10T
						5941457	G/A	4A/13G	0A/14G	5944381	T/A	11A/4T	3A/10T
Glyma12g08230	5980455-5993620	5980786	A/C	0C/10A	6C/0A	5980716	G/A	3A/12G	0A/14G	5988175	G/C	0C/12G	1C/6G
		**5985820**	**A/T**	**0T/10A**	**5T/1A**	5980786	A/C	11C/5A	3C/11A	5988557	G/T	2T/6G	0T/9G
						**5980901**	**G/T**	**3T/12G**	**0T/14G**	5988558	T/G	1G/7T	0G/12T
						**5980962**	**A/G**	**4G/13A**	**1G/13A**	5988571	T/C	3C/11T	0C/12T
						**5981392**	**T/G**	**3G/13T**	**0G/14A**	5988679	C/A	2A/13C	0A/13C
						**5981422**	**A/G**	**2G/13A**	**0G/14A**	5988706	C/A	2A/12C	0A/13C

5981602	T/A	4A/12T	3A/11T	5988719	A/T	6T/9A	3T/11A
5981809	G/C	3C/14G	0C/14G	5989166	G/A	2A/13G	0A/14G
5981855	T/C	3C/14T	0C/14T	5989277	T/A	3A/13T	0A/14T
5981984	T/C	3C/14T	0C/14T	5989696	G/T	3T/14G	0T/13G
**5982106**	**A/T**	**3T/13A**	**0T/14A**	5989913	C/A	2A/13C	0A/14C
**5982177**	**T/G**	**3G/14T**	**0G/14T**	5990038	A/G	2G/14A	0G/14A
**5982868**	**C/A**	**3A/13C**	**0A/14C**	5990230	G/T	3T/13G	0T/12G
**5982993**	**C/A**	**4A/12C**	**0A/14C**	5990322	C/T	4T/12C	0T/12C
**5983061**	**C/G**	**5G/12C**	**3G/11C**	5990899	G/A	3A/13G	0A/14G
**5983104**	**C/G**	**0G/17C**	**2G/12C**	**5991122**	**G/A**	**1A/15G**	**0A/14G**
**5983130**	**T/A**	**2A/14T**	**0A/14T**	**5991224**	**C/A**	**3A/14C**	**0A/14C**
**5983623**	**A/T**	**3T/12A**	**0T/14A**	**5991634**	**T/C**	**3C/14T**	**0C/14T**
**5983969**	**C/T**	**3T/14C**	**0T/14C**	**5992073**	**C/T**	**12T/5C**	**3T/11C**
**5984015**	**C/T**	**0T/15C**	**2T/11C**	5992076	T/C	3C/13T	0C/14T
**5984160**	**T/C**	**3C/14T**	**0C/14T**	**5992190**	**T/G**	**3G/14T**	**0G/10T**
**5984415**	**C/A**	**2A/13C**	**0A/14C**	**5992222**	**G/T**	**2T/13G**	**0T/11G**
**5984785**	**A/C**	**7C/7A**	**3C/9A**	**5992223**	**G/T**	**2T/13G**	**0T/11G**
**5985820**	**A/T**	**2T/14A**	**3T/10A**	5992272	T/A	2A/11T	0A/10T

										5986211	G/A	7A/9G	3A/10G	**5992333**	**C/T**	**2T/12C**	**1T/11C**
										5986467	G/A	3A/4G	0A/12G	**5992340**	**C/T**	**3T/13C**	**1T/11C**
										5986563	C/A	3A/4C	0A/13C	5992563	T/A	3A/14T	0A/14T
										5986950	T/G	4G/13T	3G/11T	5992565	A/T	4T/13A	3T/11A
										5987298	C/A	5A/10C	0A/11C	**5992877**	**T/C**	**3C/13T**	**0C/14T**
										5987299	A/C	1C/13A	2C/10A	**5992902**	**G/A**	**3A/13G**	**0A/14G**
										5987767	A/G	3G/13A	0G/14A	**5993171**	**A/T**	**0T/15A**	**1T/11A**
										**5988005**	**T/C**	**3C/14T**	**0C/14T**	5993481	C/G	1G/6C	0G/12C
										5988167	A/G	2G/12A	0G/12A	5993482	A/G	1G/8A	1G/12A
Glyma15g35080	39604536-39609953	39607166	A/G	72G/182A	10G/2G	39605138	G/A	1A/9G	5A/1G	39605138	G/A	7A/9G	3A/11G	39607411	G/A	2A/15G	1A/11G
						39605584	T/C	0C/10T	3C/3T	39605455	C/T	2T/12C	3T/11C	**39607944**	**T/A**	**13A/3T**	**7A/7T**
						39606116	T/A	2A/8T	6A/0T	39605605	C/T	1T/6C	1T/10C	39608678	C/A	2A/14C	2A/11C
						39606221	C/T	0T/10C	3T/3C	39605606	A/T	2T/6A	1T/9A	39608753	T/A	3A/13T	4A/10T
						39606561	C/A	1A/9C	1A/5C	39605608	G/T	1T/7G	0T/10G	39608760	G/T	2T/14G	2T/12G
						39606988	G/T	1T/9G	2T/4G	39605612	C/G	1G/11C	0G/13C	39608859	C/T	1T/13C	1T/12C
						39607031	A/T	1T/9A	4T/2A	39605664	T/C	2C/11T	1C/13T	39608958	A/G	0G/12A	1G/10A
						39607090	A/T	2T/8A	6T/0A	39605973	C/T	6T/9C	4T/9C	39608959	A/G	2G/12A	0G/10A
						39607298	C/T	7T/3C	6T/0C	39606068	A/G	2G/15A	1G/13A	39608965	A/T	10T/5A	8T/2A

		39607359	C/T	1T/9C	1T/5C	39606116	T/A	14A/3T	6A/7T	39608983	A/T	1T/15A	0T/12A
		39607944	T/A	2A/8T	6A/0T	39606561	C/A	5A/11C	3A/11C	39609048	A/G	3G/14A	2G/11A
		39608099	T/A	0A/10T	3A/3T	39606730	A/G	2G/14A	2G/12A	39609101	A/G	3G/13A	3G/11A
		39608760	G/T	1T/9G	1T/5G	39606765	T/C	3C/11T	3C/10T	39609176	A/T	1T/15A	1T/13A
		39608914	G/C	0C/10G	3C/3G	39606826	A/C	2C/15A	0C/13A	39609291	A/T	2T/14A	0T/14A
		39609048	A/G	1G/9A	1G/5A	39606873	C/A	2A/14C	0A/14C	39609489	A/T	1T/12A	1T/13A
		39609137	A/G	0G/10A	3G/3A	39606929	A/G	0G/16A	2G/12A	39609498	G/A	1A/8G	0A/11G
		39609702	T/A	1A/9T	1A/5T	39606988	G/T	6T/10G	4T/9G	39609521	T/C	0C/17T	2C/12T
		39609744	T/G	1G/9T	2G/4G	39607031	A/T	5T/12A	3T/11A	39609576	C/A	3A/14C	2A/12C
		39609846	A/C	0C/10A	3C/3A	39607166	A/G	5G/11A	1G/12A	39609702	T/A	3A/14T	2A/11T
						39607211	C/T	2T/13C	0T/14C	39609744	T/G	6G/10T	4G/10T
						39607298	C/T	16T/0C	9T/3C				
Glyma16g26030	30193624-30194977	30193928	C/T	0T/10T	3T/3C	30193671	A/T	8T/9A	3T/8A	**30194050**	**C/A**	**2A/7C**	**2A/7C**
		30194803	A/C	3C/7A	6C/0A	**30194022**	**C/A**	**2A/6C**	**1A/7C**	**30194077**	**A/C**	**2C/8A**	**1C/9A**
						**30194029**	**T/G**	**2G/6T**	**0G/7T**	**30194175**	**A/C**	**1C/5A**	**0C/1A**
						**30194032**	**C/G**	**1G/9C**	**0G/9C**	**30194394**	**G/C**	**1C/14G**	**4C/9G**
						**30194034**	**G/C**	**2C/8G**	**0C/9G**	30194803	A/C	7C/9A	4C/10A
						**30194036**	**G/C**	**1C/8G**	**1C/7G**	30194916	G/C	3C/13G	0C/13G

Table S3. Insertions in the coding (bold) and non-coding sequences of three candidate genes of flowering time or seed size in wild and cultivated soybeans

Gene		Position of Insertion (bp)	No of bases	Base
Name	Position (bp)			
Glyma12g08150	5874276-5880441	5875446	153	ATAGGGAAACACGAGTTACCTCATTCCTATTCCTATGACAGCAAAATCGCTGACCCAATTTCCCACTTAATTTAAAAGCAAAGCAGAAATAATTCTTAACTTGAGCATCAGGGATTTTAAAAGAAAAAAAAGAAAGAAGAAAACGACAAAGAA
		5875967	1	T
		5878444	252	ATTTTCTTATCTTTGGCAGGACAAATGAGATAAAAAGATAATTTGACTATTATGCAATAATAAAGTAAGAAGATTCCTTCTTATGAAAACTGAATACATCCTTGAAAACAGGTTTTATACCACTTTTGGTTCTTTGCGTGCTTGTTATCTCTTTAGGATTTGAACCTAATTTTAGTTTACAACAATAATTTTCTACCTTAACGATTTCCTATGAGATAGGATTGCAATCCAAGAAAACATCCTTTACCAAGG
**Glyma12g08210**	**5937860-5944400**	**5938472**	**321**	**GTAAGTTCTACTTTCTAACTAACATTAGAGCATGGAGGCTTACAAACAAAAACATGGTTAATTGCTATGAATGCTGTACAGCAATAAAAATGTTTTAGGCTCATCATTTTATCTAGAAACTATGGGACATTATAACATAAAATAACCAGCCGAAGAATACTGATATTCCAAGATTAGGTACAACCTCCTTGAAAAATGCAGAGTCTGCTTCTGGCCCTCCTTGGCATGAAATTAAAATCAAGGAACTGCTAGTAAAAAAAAACCAACTTACCTGTGATATGATAAATTGGAGAATTTATTGCATATTTTGTTTCTGGCACT**
		**5939365**	**470**	**CCAGTGTATGTAATTAAGGTAACAAGGGTAGGAAGGAACAAATGCTGGGTTGGCCAACAAACATCAGAGTCAAAGTACCATCATAGTTTCCACCTACAATTCTGCAAAGTTTGAGATTTTGGAGATGGAAGAAGAGGGTTTTGGTGCAAATTAAAGAAAGTCGAAGCAGCAAAACAAAGCAAAACAAAAAGAAAGAAAGCTATGATTGGCTAATAACTAAAGCAAGAGCAATTTTGAGCTGTGGCTTCATTTATTCCAACCCATACACTGAAAATAAAAGAAAGGAAACCATTTAGTTCCATACAAACACTTGAATAATAATGCTCCTCGTGCTATGAAACAAGCCACTTATGTTTGATGATTTCCCGAGACAATAAATTCATCTTCAGAACCTAAAGAAATCATGAACAGATGATGAGCAACATGTAGTAGCTACATCGTTTCAGATACCAGATAGCGAGAGAGGGAGA**
Glyma12g08230	5980455-5993620	5993482	1	G

The results were based on sequence data of Lam *et al*. (2010).

Table S4. Comparison of domestication genes detected in this study with domestication QTL (or genes) previously reported

Domestication and association analyses in this study				Domestication gene detected by Chung et al. 2014	Public QTL around domestication gene		
Chr	Loci	Domestication gene	Trait associated		Marker associated	Traits	Reference*
Gm11	10957940	*Glyma11g15310,Glyma11g15330*	Seed size		sat_128-satt519	seed weight 20-1, 20-3, 20-4	Lian et al. 2010
Gm11	11111516	*Glyma11g15480*	Seed size		sat_128-satt519	seed weight 20-1, 20-3, 20-4	Lian et al. 2010
Gm11	15362036	*Glyma11g18720*	Flowering time		satt519-satt583	reproductive stage length 8-1	Komatsu et al. 2012
Gm12	5895633	*Glyma12g08150,Glyma12g08160*	Flowering time	*Glyma12g08150,Glyma12g08160*	satt442	reproductive stage length 7-3	Li et al. 2008
Gm12	5895655	*Glyma12g08150,Glyma12g08160*	Flowering time	*Glyma12g08150,Glyma12g08160*	satt442	reproductive stage length 7-3	Li et al. 2008
Gm12	5895785	*Glyma12g08150,Glyma12g08160*	Flowering time	*Glyma12g08150,Glyma12g08160*	satt442	reproductive stage length 7-3	Li et al. 2008
Gm12	5957099	*Glyma12g08210,Glyma12g08230*	Flowering time	*Glyma12g08210,Glyma12g08230*	satt442	reproductive stage length 7-3	Li et al. 2008
Gm16	30207175	*Glyma16g26030,Glyma16g26050*	Seed size		satt215, satt547	seed yield 23-2, 23-3	Guzman et al. 2007
Gm16	30210113	*Glyma16g26030,Glyma16g26050*	Seed size		satt215, satt547	seed yield 23-2, 23-3	Guzman et al. 2007
Gm17	2040047	*Glyma17g04080*		*Glyma17g04080*			
Gm17	2690293	*Glyma17g04080*		*Glyma17g04080*			
Gm17	3193537	*Glyma17g04760,Glyma17g04770*		*Glyma17g04760,Glyma17g04770*			

* References were as follows.

Chung W H, *et al*. 2014. Population structure and domestication revealed by high-depth resequencing of Korean cultivated and wild soybean genomes. *DNA Res* **21:** 153–167.

Guzman P S, *et al*. 2007. QTL associated with yield in three backcross-derived populations of soybean. *Crop Sci*. **47**:111-122.

Komatsu K, *et al*. 2012. Identification of QTL controlling post-flowering period in soybean. *Breed. Sci.* **61**(5):646-652.

Li D D, *et al*. 2008. Soybean QTL for yield and yield components associated with *Glycine soja* alleles. *Crop Sci*. **48**:571-581.

Lian Q, *et al*. 2010. QTL analysis of root traits as related to phosphorus efficiency in soybean. *Ann. Bot.* **106**(1):223-234.

Table S5. Ecological regions of 286 soybean cultivars in China.

NO.	Group	Origin	Ecological region	NO.	Group	Origin	Ecological region	NO.	Group	Origin	Ecological region
001	Landrace	Jiangsu	MLC	043	Landrace	Liaoning	NRT	085	Landrace	Guizhou	SWP
002	Landrace	Jiangsu	MLC	044	Landrace	Liaoning	NRT	086	Landrace	Fujian	CTS
003	Landrace	Jiangsu	MLC	045	Landrace	Liaoning	NRT	087	Landrace	Guizhou	SWP
004	Landrace	Anhui	MLC	046	Landrace	Liaoning	NRT	088	Landrace	Guizhou	SWP
005	Landrace	Shandong	HHH	047	Bred	Liaoning	NRT	089	Landrace	Shaanxi	MLC
006	Landrace	Shandong	HHH	048	Landrace	Liaoning	NRT	090	Landrace	Shaanxi	MLC
007	Bred	Shandong	HHH	049	Landrace	Liaoning	NRT	091	Landrace	Shaanxi	MLC
008	Landrace	Zhejiang	MLC	050	Landrace	Liaoning	NRT	092	Landrace	Shaanxi	MLC
009	Bred	Jiangsu	HHH	051	Landrace	Zhejiang	MLC	093	Landrace	Shaanxi	MLC
010	Landrace	Fujian	CTS	052	Bred	Liaoning	NRT	094	Landrace	Shaanxi	MLC
011	Landrace	Fujian	CTS	053	Landrace	Zhejiang	MLC	095	Landrace	Shaanxi	MLC
012	Landrace	Fujian	CTS	054	Landrace	Beijing	HHH	096	Landrace	Shaanxi	MLC
013	Landrace	Fujian	CTS	055	Landrace	Fujian	CTS	097	Landrace	Shaanxi	MLC
014	Landrace	Fujian	CTS	056	Landrace	Fujian	SCT	098	Landrace	Shaanxi	MLC
015	Landrace	Jiangsu	MLC	057	Landrace	Fujian	CTS	099	Landrace	Shaanxi	MLC
016	Bred	Hainan	SCT	058	Landrace	Hunan	CTS	100	Landrace	Shaanxi	MLC

017	Landrace	Shanghai	MLC	059	Landrace	Hunan	CTS	101	Landrace	Shaanxi	MLC
018	Bred	Heilongjiang	NRT	060	Bred	Hunan	CTS	102	Bred	Jiangsu	MLC
019	Landrace	Guangdong	SCT	061	Landrace	Zhejiang	MLC	103	Bred	Hubei	MLC
020	Landrace	Guangdong	SCT	062	Landrace	Zhejiang	CTS	104	Landrace	Hubei	MLC
021	Landrace	Guangdong	SCT	063	Landrace	Zhejiang	MLC	105	Landrace	Hubei	MLC
022	Landrace	Guangdong	SCT	064	Landrace	Zhejiang	CTS	106	Landrace	Hubei	MLC
023	Landrace	Guangdong	SCT	065	Landrace	Zhejiang	CTS	107	Landrace	Hubei	MLC
024	Landrace	Guangdong	SCT	066	Landrace	Zhejiang	CTS	108	Landrace	Hubei	MLC
025	Landrace	Guangdong	SCT	067	Landrace	Zhejiang	MLC	109	Landrace	Hubei	MLC
026	Landrace	Guangdong	SCT	068	Landrace	Zhejiang	MLC	110	Landrace	Hubei	MLC
027	Landrace	Guangdong	SCT	069	Landrace	Zhejiang	CTS	111	Landrace	Jiangxi	MLC
028	Landrace	Guangdong	SCT	070	Landrace	Zhejiang	CTS	112	Landrace	Jiangxi	CTS
029	Landrace	Guangdong	SCT	071	Landrace	Zhejiang	CTS	113	Landrace	Jiangxi	CTS
030	Landrace	Guangdong	SCT	072	Landrace	Zhejiang	CTS	114	Landrace	Shandong	HHH
031	Landrace	Guangdong	SCT	073	Landrace	Zhejiang	MLC	115	Landrace	Shandong	MLC
032	Bred	Guangdong	SCT	074	Landrace	Zhejiang	CTS	116	Landrace	Hunan	CTS
033	Landrace	Guangdong	SCT	075	Landrace	Zhejiang	CTS	117	Landrace	Yunnan	SCT
034	Landrace	Guangdong	SCT	076	Landrace	Zhejiang	CTS	118	Landrace	Yunnan	SWP

035	Landrace	Guangdong	SCT	077	Landrace	Zhejiang	CTS	119	Bred	Jiangsu	HHH
036	Landrace	Guangdong	SCT	078	Landrace	Zhejiang	MLC	120	Bred	Anhui	HHH
037	Landrace	Liaoning	NRT	079	Landrace	Hunan	CTS	121	Landrace	Hunan	CTS
038	Landrace	Liaoning	NRT	080	Landrace	Zhejiang	MLC	122	Landrace	Guizhou	SWP
039	Landrace	Fujian	MLC	081	Landrace	Zhejiang	CTS	123	Landrace	Guangxi	SWP
040	Landrace	Liaoning	NRT	082	Landrace	Anhui	MLC	124	Landrace	Guangxi	SCT
041	Landrace	Liaoning	NRT	083	Landrace	Anhui	MLC	125	Bred	Anhui	MLC
042	Landrace	Liaoning	NRT	084	Landrace	Guizhou	SWP	126	Landrace	Jiangsu	MLC
127	Landrace	Jiangsu	MLC	171	Bred	Anhui	HHH	215	Bred	Jiangsu	MLC
128	Landrace	Hubei	MLC	172	Bred	Henan	HHH	216	Bred	Jiangsu	MLC
129	Landrace	Hubei	MLC	173	Bred	Henan	HHH	217	Bred	Beijing	HHH
130	Bred	Jiangsu	MLC	174	Bred	Henan	HHH	218	Bred	Shandong	HHH
131	Landrace	Jiangsu	MLC	175	Bred	Henan	HHH	219	Bred	Beijing	HHH
132	Landrace	Heilongjiang	NRT	176	Bred	Henan	HHH	220	Bred	Henan	HHH
133	Landrace	Jiangsu	MLC	177	Bred	Henan	HHH	221	Bred	Henan	HHH
134	Bred	Jiangsu	MLC	178	Bred	Henan	HHH	222	Bred	Henan	HHH
135	Bred	Shandong	HHH	179	Bred	Henan	HHH	223	Bred	Shandong	HHH
136	Landrace	Jiangsu	MLC	180	Bred	Henan	HHH	224	Bred	Shandong	HHH

137	Bred	Anhui	HHH	181	Bred	Hebei	HHH	225	Bred	Henan	HHH
138	Bred	Beijing	HHH	182	Landrace	Beijing	HHH	226	Bred	Hebei	HHH
139	Bred	Beijing	HHH	183	Bred	Beijing	HHH	227	Bred	Henan	HHH
140	Bred	Heilongjiang	NRT	184	Landrace	Anhui	MLC	228	Bred	Shandong	HHH
141	Landrace	Heilongjiang	NRT	185	Bred	Anhui	HHH	229	Bred	Xuzhou	HHH
142	Bred	Shandong	HHH	186	Bred	Henan	HHH	230	Bred	Shandong	HHH
143	Bred	Henan	HHH	187	Bred	Henan	HHH	231	Bred	Jiangsu	HHH
144	Landrace	Hubei	MLC	188	Bred	Henan	HHH	232	Bred	Shandong	HHH
145	Landrace	Liaoning	NRT	189	Landrace	Henan	HHH	233	Bred	Shandong	HHH
146	Landrace	Sichuan	CTS	190	Bred	Henan	HHH	234	Bred	Shandong	HHH
147	Landrace	Sichuan	CTS	191	Landrace	Henan	HHH	235	Bred	Beijing	HHH
148	Landrace	Sichuan	SWP	192	Bred	Shandong	HHH	236	Bred	Hebei	HHH
149	Landrace	Sichuan	SWP	193	Bred	Shandong	HHH	237	Bred	Shandong	HHH
150	Landrace	Sichuan	CTS	194	Bred	Shandong	HHH	238	Bred	Shandong	HHH
151	Landrace	Sichuan	SWP	195	Bred	Shandong	HHH	239	Bred	Shandong	HHH
152	Landrace	Sichuan	SWP	196	Bred	Shandong	HHH	240	Bred	Shanxi	HHH
153	Bred	Anhui	MLC	197	Bred	Shandong	HHH	241	Bred	Jiangsu	HHH
154	Bred	Shanxi	HHH	198	Bred	Fujian	CTS	242	Bred	Henan	HHH

155	Bred	Heilongjiang	NRT	199	Bred	Fujian	SCT	243	Bred	Shandong	HHH
156	Bred	Heilongjiang	NRT	200	Bred	Shanxi	HHH	244	Bred	Shandong	HHH
157	Landrace	Zhejiang	CTS	201	Landrace	Guangxi	SWP	245	Bred	Shandong	HHH
158	Landrace	Zhejiang	CTS	202	Bred	Fujian	CTS	246	Bred	Shandong	HHH
159	Landrace	Zhejiang	CTS	203	Landrace	Fujian	CTS	247	Bred	Shandong	HHH
160	Bred	Beijing	HHH	204	Bred	Shandong	HHH	248	Bred	Anhui	HHH
161	Landrace	Beijing	HHH	205	Bred	Liaoning	NRT	249	Bred	Anhui	MLC
162	Bred	Anhui	MLC	206	Bred	Shandong	HHH	250	Bred	Shandong	HHH
163	Landrace	Fujian	SWP	207	Landrace	Guangxi	SWP	251	Bred	Shandong	HHH
164	Landrace	Sichuan	SWP	208	Bred	Heilongjiang	NRT	252	Bred	Anhui	HHH
165	Landrace	Sichuan	SWP	209	Bred	Liaoning	NRT	253	Bred	Beijing	HHH
166	Landrace	Sichuan	SWP	210	Landrace	Guangxi	SCT	254	Bred	Shandong	HHH
167	Landrace	Sichuan	SWP	211	Landrace	Shandong	HHH	255	Bred	Jiangsu	HHH
168	Landrace	Yunnan	SCT	212	Landrace	Yunnan	SCT	256	Bred	Xuzhou	HHH
169	Bred	Shandong	HHH	213	Landrace	Jiangsu	MLC	257	Bred	Hebei	HHH
170	Landrace	Shandong	HHH	214	Wild	Liaoning	NRT	258	Bred	Anhui	HHH
259	Bred	Shandong	HHH	269	Wild	Jilin	NRT	279	Bred	Hebei	HHH
260	Wild	Jiangsu	MLC	270	Wild	Neimenggu	NRT	280	Bred	Shandong	HHH

261	Wild	Jiangsu	MLC	271	Wild	Jilin	NRT	281	Bred	Anhui	HHH
262	Wild	Jiangsu	MLC	272	Wild	Jiangsu	MLC	282	Bred	Anhui	HHH
263	Wild	Hubei	MLC	273	Bred	Jiangsu	MLC	283	Bred	Henan	HHH
264	Wild	Hubei	MLC	274	Bred	Jiangsu	MLC	284	Bred	Henan	HHH
265	Wild	Hubei	MLC	275	Bred	Jiangsu	MLC	285	Bred	Shandong	HHH
266	Wild	Hubei	MLC	276	Landrace	Jiangsu	MLC	286	Bred	Henan	HHH
267	Wild	Hubei	MLC	277	Bred	Henan	HHH				
268	Wild	Hubei	MLC	278	Bred	Henan	HHH				

Note: Six ecological regions were as follows.

Northern single cropping, spring planting eco-region(NRT): 28;

Huanghuaihai double cropping, spring and summer planting eco-region (HHH): 101;

Middle and lower Changjiang valley double cropping, spring and summer planting eco-region(MLC): 75;

Central south multiple cropping, spring, summer and autumn planting eco-region(CTS): 38;

Southwest plateau double cropping, spring and summer planting eco-region(SWP): 18;

South China tropical multiple cropping, all season planting eco-region(SCT): 26.
